# ZEB1 stratifies the response to Sorafenib and Mdivi-1 combination therapy in hepatocellular carcinoma

**DOI:** 10.1038/s41598-025-16379-6

**Published:** 2025-08-19

**Authors:** H. Freudenstein, M. Strecker, S. Gylstorff, W. Shi, M. Boettcher, S. Medunjanin, C. Catapano, C. Siba, C. Wex, T. Wartmann, A. Y. Sanin, M. Franz, J. Arend, D. Mougiakakos, M. Pech, R. S. Croner, U. D. Kahlert, F. Stelter

**Affiliations:** 1 Molecular and Experimental Surgery, Clinic for General-, Visceral-, Vascular-, and Transplantation Surgery, Medical Faculty, University Medical Center Magdeburg, Magdeburg, Germany; 2 Experimental Radiology, University Clinic for Radiology and Nuclear Medicine, Medical Faculty, University Medical Center Magdeburg, Magdeburg, Germany; 3 Department of Hematology and Oncology, Otto-von-Guericke University Magdeburg, Magdeburg, Germany; 4 Clinic for Experimental Cardiology and Angiology, Medical Faculty, University Medical Center Magdeburg, Magdeburg, Germany; 5 Institute of Oncology Research (IOR), Università della Svizzera Italiana, Bellinzona, Switzerland; 6 Research Campus Stimulate, University Magdeburg, Magdeburg, Germany

**Keywords:** Hepatocellular carcinoma, ZEB1, EMT, Mitochondrial metabolism, Sorafenib, Mdivi-1, Cancer metabolism, Cancer therapeutic resistance, Preclinical research, Liver cancer

## Abstract

Primary liver cancer is one of the most frequently diagnosed and deadliest cancers. Zinc finger E-box binding homeobox 1 (ZEB1) is negative prognostic factor in liver cancer by promoting therapy resistance and tumorigenesis. Interfering in pathways of cellular metabolism emerges as a potent strategy to overcome tumor cells resistance to therapy. Our study aims to investigate the apoptotic potential of pharmacological inhibition of mitochondrial metabolism in primary liver cancer and whether this strategy can reduce ZEB1-associated resistance to standard of care chemotherapy Sorafenib. ZEB1 mRNA levels in patient samples and cancer cell lines were computationally screened using public datasets. Transcript and protein abundance of ZEB1 and regulators of mitochondrial fission and fusion were quantified in patient-matched tumor and non-tumor tissues of hepatocellular carcinoma (HCC) and cholangiocellular adenocarcinoma (CCA) from our clinic and common liver cancer cell lines. Functional assays on cell models with varying ZEB1 expression exposed to mitochondrial division inhibitor Mdivi-1 included mitochondrial mass quantification, mitochondrial membrane potential examination, apoptosis, extracellular flux, and cell growth analyses. Mdivi-1 treatment in human-physiological achievable concentration effectively induced apoptosis in all tested cell models. ZEB1 expression was heightened in younger patients, and dynamin-related protein 1 (Drp1) protein abundance was elevated in a subgroup of tumor tissue compared to healthy tissue. Cancer cell lines with high ZEB1 expression showed increased mitochondrial fission marker Drp1 and larger total mitochondrial mass, preserved membrane potential with reduced mitochondrial ATP production and respiration, resulting in an overall reduced mitochondrial fitness. Pharmacological inhibition of mitochondrial fission using Mdivi-1 reduced HCC resistance to Sorafenib in ZEB1-driven liver cancer. A subset of HCC cell lines with elevated ZEB1 levels exhibit increased mitochondrial mass and reduced metabolic activity. Targeting mitochondrial division in HCC by treatment with Mdivi-1 in combination with Sorafenib demonstrates a synergistic therapeutic effect in hepatocellular carcinoma (HCC) cell lines characterized by high ZEB1 expression. Further in vivo validation is needed to confirm these findings and evaluate this potential combined treatment option.

**Supplementary Information:**

The online version contains supplementary material available at 10.1038/s41598-025-16379-6.

## Introduction

Primary liver cancer is a significant contributor to cancer-related fatalities worldwide, constituting more than 800.000 deaths worldwide (2023)^1^. The five-year survival rates, ranging from 5 to 30%, are generally lower when compared to other gastrointestinal malignancies, like colorectal cancer, where survival rates are as high as 62%^2^. The most common primary liver malignancies are hepatocellular carcinoma (HCC) and cholangiocellular adenocarcinoma (CCA). With up to 90% of all cases HCC is the more prevalent type. One of the main risk factors for primary liver malignancies are chronic liver diseases such as fibrosis or cirrhosis caused by obesity, viral infection and alcohol abuse. Therefore, the treatment of primary liver cancer remains a major obstacle, especially in affluent western societies^3,4^. Common therapy modalities in primary liver malignancies include surgical resection, local ablative therapies (chemoembolization or radiation), targeted therapies and checkpoint inhibitors^5,6^. For both HCC and CCA therapeutic options in advanced stages and upon tumor recurrence are limited. There is a need to explore further biomarkers for patient-matched precision medicine.

The significance of ZEB1 as a driving force in tumorigenesis across different cancer types, including HCC and CCA, has been extensively reported. ZEB1 not only drives EMT and promotes metastasis but also plays a crucial role in the earlier stages of disease by regulating key cellular processes related to differentiation and proliferation^7–9^. In HCC it has been linked to lower survival rates, higher recurrence and generally more invasive forms of disease^10–12^. Similar findings regarding CCA exist, yet research in this area remains limited^13^.

Metabolic reprogramming and changes especially in mitochondrial metabolism are crucial for tumor progression and metastasis and are widely discussed in the context of both CCA and HCC. In primary liver cancer, epithelial-mesenchymal transition (EMT) and mitochondrial fission - shortened and fragmented mitochondria - have been linked to tumorigenesis and progression^14–16^. The involvement of ZEB1 as an EMT marker in metabolism has been examined, particularly in relation to glycolysis and Warburg effect. Zhou et al. demonstrated that ZEB1 promotes the Warburg effect in hepatocellular carcinoma a phenomenon that has also been observed in other malignancies, including breast cancer^17,18^. Meanwhile, research on ZEB1’s role in mitochondrial metabolism and dynamics, governed by the proteins Drp1 and Mitofusin-2 (Mfn2) is limited but has been subject of recent research. Current findings indicate a suppression of mitochondrial fusion in hematopoietic stem cells exhibiting a high ZEB1 expression. ZEB1 transcriptionally represses Mfn-2, sustaining an immature mitochondrial state^19^.

Our study seeks to address a knowledge gap by presenting ZEB1 as a biomarker associated with elevated Drp1 levels, thereby directly influencing mitochondrial fission in hepatocellular carcinoma cell lines. To validate our gene of interest and provide more potential targets in ZEB1’s influence on mitochondrial metabolism we are using extensive datasets for robust bioinformatic analysis, focusing on the earlier mentioned key role of the liver in lipid and energy metabolism. Furthermore, we are conducting a comparative analysis of total ZEB1 and Drp1 protein and RNA expression levels in donor-matched liver tumor and non-tumor liver tissues obtained from Western Caucasian individuals. We aim to utilize this new understanding to explore potential clinical applications through combined chemotherapeutic strategies, specifically pairing Sorafenib, a guideline-recommended treatment, with inhibitor of mitochondrial fission.

## Methods

### Bioinformatic assessment

We obtained transcriptome data for HCC and CCA from the The Cancer Genome Atlas (TCGA) database. Subsequently, we extracted markers related to ZEB1, citrate cycle, and fatty acid metabolism to create a consolidated expression matrix. Spearman’s method was applied to analyze the correlation between ZEB1 and the other markers individually. We considered correlations with an absolute coefficient > 0.1 and a p-value < 0.05 to be statistically significant. The full data set can be reviewed in (Supplementary Fig. 3). Copyright license has been obtained for the KEGG database^20,21^.

### Patient sample acquisition

The visceral medicine biobank study for the elucidation of molecular mechanisms in the development and progression of visceral medical and /or infectious diseases was approved by the ethics committee of the University Hospital Magdeburg (#47/22) in accordance with the Declaration of Helsinki. Informed consent was obtained from all subjects and/or their legal guardian(s) before tissue acquisition. We obtained a total of 15 patient samples for our study, which were separated for tumor and non-tumor areas by a German-board certified pathologist. The clinical parameters of the patients are listed in (Table 1). We acquired tissue from the operating room of the Department of Visceral Surgery or from biopsies in collaboration with the Department of Radiology.

**Table 1:** Clinical data of tumor patients in our cohort.

Tumor code	Age	Gender	Tumor entity	Tumor	Grading	ZEB1	Histo- and molecular Pathology	Clinical outcome
HCC-1	70	Male	HCC	pT1b L0 V0 Pn0 R0	G2		Child A cirrhosis, no AFP	No recurrence, received liver transplantation after 3 years, neoadjuvant TACE
HCC-2	72	Male	HCC	pT1b pN0 (0/10) L0 V0 Pn0 R0	G2		AFP neg	No recurrence since 2 years
HCC-3	76	Male	HCC	pT2 L0 V1 Pn0 R0	G2		AFP pos	peritoneal tunor recurrence 3 months after surgery, start Atezolizumab + Bevacicumab, stable disease for 2 years
HCC-4	73	Male	HCC	pT2 V1 R0	G2		Child A cirrhosis	loss of follow up
HCC-5	56	Male	HCC	pT2 pN0(0/1) V1 Pn0	G2	+	initially AFP neg, recurrence AFP pos	10 months after surgery lung metastasis and local HCC recurrence. AtezoBev
CCA-1	60	Female	CCA	pT4 pN0(0/25) L0 V1 Pn1 R1	G3		Child A cirrhosis	Adjuvant therapy with capecitabine, local tumor recurrence after 18 months, surgical resection, complete remission for following 12 months
CCA-2	34	Female	CCA	pT3 pN1(5/8) L1 V1 Pn1 R1	G3		FGFR2 mutation, P53 mutation	Died of postop. liver failure, no follow up data
CCA-3	59	Female	CCA	ypT2 ypN1(1/1) L1 V1 Pn0 R0 M0	G3	+	KRAS mutation	Adjuvant therapy Gem/Cis/Durva, afterwards Capeticabine, 2 years later local recurrence liver - Brachytherapy, 8 month later multiple local recurrence, lung metastasis, further systemic chemotherapy

### In vitro models

Cell lines Huh7, HepG2, HuCC-T1 and HuCC-A1 were originally purchased from ATCC and kindly provided to us by the V Keitel-Anselmino, Department of Gastroenterology, University Medical Center Magdeburg. Carlo Catapano of the Institute of Oncology Research, Bellinzona, Switzerland, kindly provided Snu475, Snu378 and Snu423.

All cell lines were cultured in a 37 °C humid incubator containing 5% CO2 using RPMI supplemented with 10% Fetal bovine serum and 1% Pen/strep, except for HuCC-A1 cell line, which was cultured in Ham’s F12 Medium (+ 10% FBS, + 1% PS) and HepG2 cell line, which was cultured in Dulbecco modified Eagle’s Medium (DMEM) + 10% FBS, 1% P/S and 1% Sodium Pyruvate. Cell growth was monitored daily and split when necessary.

Characteristics of cell lines used are outlined in Supplementary Table S3.

### Real-time polymerase chain reaction analysis

Cell pellets were collected after trypsinizing with 0.05% Trypsin for 10–15 min and thorough washing with culture media and Phosphate-Buffered Saline. Organoids were harvested and brought to single cell level by mechanical dissociation and TrypLE treatment for 30 min. RNA was isolated using the Qiagen RNAeasy Minikit (cat. No. 74104) following the manufacturer’s instructions and concentrations were determined photometrically. Subsequently, reverse transcription was performed using 1 µg of RNA and the New England BioLabs LunaScript^®^ RT Super Mix (cat. No. M3010) according to manufacturer’s instruction. Primers used in this study can be found in Supplementary Table S2.

We employed the Luna Universal qPCR Master Mix (cat. No. M3003L) in the qPCR reaction, following the manufacturer’s instructions. The BioRad CFX96 PCR detection system was utilized, and statistical analysis was carried out using the ddCT method.

### Western blot analysis

Cells were harvested in a lysis buffer (1.5% Triton X-100, 50 mM HEPES, 150 mM NaCl, 1,5 mM MgCl_2_, 10% Glycerin, 5 mM EDTA, 10 mM Na_4_P_2_O_7_). After sonicating the lysate three times for five seconds each, it was centrifuged at 15.000 g for 20 min at 4 °C. For electrophoresis 20 µg of protein was denatured at 95 °C for 5 min and separated in 7.5% SDS-Page at 85 V for 60 min and transferred to a Nitrocellulose membrane using the Bio-Rad Wet/Tank Blotting system and Towbin Buffer (25 mM Tris, 192 mM Glycin, 20% Methanol, 0.1% SDS). Blotting was performed at 50 V for 100 min at 4 °C. Subsequently, blocking was performed in 5% milk in TBS-T buffer. Primary antibodies were incubated at 4 °C overnight, secondary antibodies and actin primary antibody were performed at room temperature for one hour. Proteins were visualized by enhanced chemo luminescence (ECL) and fluorescence. The concentrations of antibodies are detailed in (Supplementary Table 1). Drp1 expression was compared to the sample with the lowest Drp1 expression. Fiji (ImageJ) was used for quantification as. Briefly, images were converted to grayscale and bands were selected with the rectangular selection tool. The background was subtracted, and each band was measured. The same process was carried out for beta-actin, whereas the most prominent band was set as the 100% value. Drp1 expression data was then normalized by dividing the Drp1 expression data relative to the sample with the lowest expression by the relative intensity of the corresponding beta-actin band. The expression values were then transferred to GraphPad Prism for statistical evaluation by Wilcoxon signed-rank test for non-parametric paired data.

### Pharmacological treatment assays

To analyse combination effects, cells were treated with Mdivi-1 and Sorafenib and the Combination Index (CI) was calculated as previously described using the program CompuSyn^22^. In order to determine IC50 concentrations for our combination treatment experiments, we used CellTiter-Glo 2.0 Luminsecent cell viability assay (cat. No. G7571). 3.000 cells were seeded in triplicates onto Greiner flat white 96 well plates in 100 µl of media. After 24 h Sorafenib and/or Mdivi-1 were added to the cells in concentrations commonly used in literature^23^. (Sorafenib: 1 µM, 3 µM, 5 µM, 7 µM, 12 µM, Mdivi-1: 1 µM, 10 µM, 20 µM, 40 µM, 100 µM). Viability was photometrically assessed after 72 h and the IC50 was determined graphically, which subsequently formed the foundation for the trial on combination therapy. The IC50 for Mdivi-1 was established at 60 µM across all cell lines. The IC50 for Sorafenib was calculated for each cell line individually: Huh7: 4 µM, HepG2: 6 µM, Snu387: 10 µM, Snu475: 10 µM.

For combination therapy treatment, 3.000 cells were seeded in a Greiner flat white 96 well plate. After 24 h, they were treated with 6 different concentrations (including a vehicle control: 0.25 × 0.5x, 1x, 2x, 4x IC50) of Sorafenib and Mdivi-1 in combination and monotherapy, in two technical replicates each. Cell viability was assessed using CTG after 72 h. The viability values for each concentration were finally divided by the vehicle control and the quotient subsequently used to calculate the combination Index as previously described. The according pipette scheme and calculation of the Combination Index were previously described by Chou et al.^24^ In the analysis, CI < 1 is defined as synergism, CI = 1 as additive effect and CI > 1 as antagonism.

### MitoTracker^®^ analysis

MitoTracker Deep Red was purchased from Invitrogen (cat. No. M46753) and used according to manufacturer’s instructions. Flow cytometry values were assessed using the BD FACSCanto™ II. We conducted data analysis using floreada.io and compared mean fluorescence intensity (MFI). Mean fluorescence intensity is defined as the ratio of fluorescence intensity stained divided by fluorescence intensity of the unstained control.

### JC-1 mitochondrial membrane potential and Annexin V apoptosis assay

Cells were seeded at a density of 250.000 cells per 6-well 24 h prior to the beginning of pharmacological treatment with MDIVI-1. Pharmacological treatment was conducted at an IC50, as described above, for 48 h in all cell lines. JC-1 was purchased form Hycultec (HY-15534) and Annexin V from BioLegend (cat. No. 640932). After harvesting the cells JC-1 staining was performed at 37 °C at a working concentration of 2 µM for 30 min. Annexin V was combied with ZombieViolet (BioLegend cat. No. 423113) and incubated according to manufacturer ‘s instructions.

Mitochondrial membrane potential was assessed using JC-1 staining, with cells exhibiting high red fluorescence (JC-1 aggregates) considered to have intact mitochondrial membrane potential, while increased green fluorescence (JC-1 monomers) indicated depolarization. Apoptosis was evaluated by Annexin V staining to detect phosphatidylserine exposure on the cell surface, combined with Zombie Violet viability dye to discriminate live from dead cells. Data analysis included gating to exclude debris and doublets, and cell populations were quantified based on Annexin V and Zombie Violet fluorescence profiles. Fluorescence was assessed using BD FACSCanto™ II. Flow cytometry data were analyzed using FlowJo software. Standardized gating strategies and appropriate controls were applied for compensation and threshold settings in FlowJo.

### Extracellular flux analysis

In our study, the concept of ‘mitochondrial fitness’ refers to the mitochondrial integrity, efficiency, and dynamic response to stress. To measure these conditions in an in vitro model, the real-time bioenergetics of the samples were determined using the XFe96 Extracellular Flux Analyzer (Seahorse, Agilent) with Wave 2.6.3, as previously described^25^. In brief, cells were seeded at a density of 15,000 cells/well in 96-well plates 24 hours prior to the analysis. For the calculation of glycolytic parameters, the extracellular acidification rate (ECAR) was recorded upon sequential injection of 10mM glucose (Sigma-Aldrich), 1 µM oligomycin (Sigma-Aldrich) and 100 mM 2-DG (Biomol). Mitochondrial parameters were calculated from oxygen consumption rate (OCR) recordings after sequential injection of 1 µM oligomycin, 1.5 µM FCCP (Sigma-Aldrich) and 3 µM of each antimycin A and rotenone (both Sigma-Aldrich). Recorded values were normalized based on the total protein content per well using a Pierce™ BCA assay protocol (Thermo Fisher). The recorded values were utilized for parameter calculations with Microsoft Excel, and graphical demonstrations as well as statistical analyses were conducted with GraphPad Prism 9.

### Statistical analysis

GraphPad PRISM software and its implemented tools (GraphPad Software, Inc., version 9, 2020) were employed for statistical analysis. Statistical analysis was conducted using the appropriate statistical method i.e. non-parametric Wilcoxon test (Mann-Whitney U Test) was used for analyzing results of Mito Tracker Analysis (Fig. 3G).

**Fig. 1:**
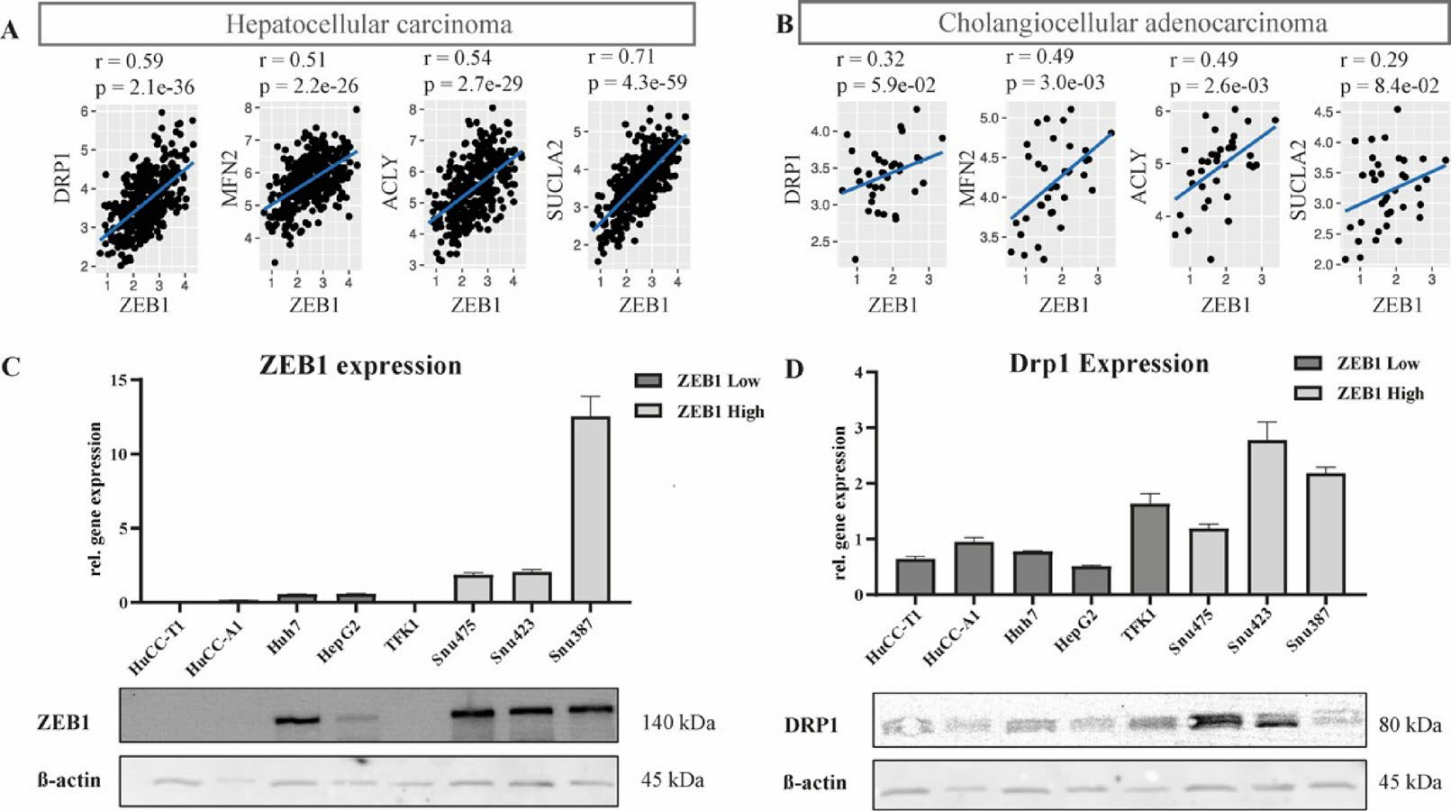
Bioinformatic analysis of ZEB1 expression correlated with genes of citric acid cycle and fatty acid metabolism, ZEB1 and Drp1 expression and protein abundance in liver cancer cells. (**A**,**B**) KEGG metabolic pathways database was used for pathway annotation and transcriptional data were extracted from The Cancer Genome Atlas, top 5 positively correlated genes in HCC and CCA. Statistical analysis was conducted using Spearman’s method. Statistically significant was defined as *p* ≤ 0.05 and effect size ‘r’ defining a medium effect from 0.3–0.49 and a large effect ≥ 0.5. (**C**) ZEB1 expression in our liver cancer cell lines on transcriptomic and protein levels. Differentiation of high and low ZEB1 cell lines was classified by at least 3-fold higher transcriptional expression of ZEB1 compared to low ZEB1 cell lines. (**D**) DRP1 expression in our liver cancer cell lines on transcriptomic and protein levels. The full western blot images can be obtained in the supplementary dataset.

## Results

### Analysis of transcriptional correlation between mitochondrial metabolism genes and ZEB1 expression in public data sets and liver cancer cell lines

To investigate the impact of ZEB1 on cellular metabolic reprogramming and potentially identify additional upstream or downstream regulators of ZEB1, we performed a computational analysis of transcriptome data from primary liver tumors in the TCGA database. Through this analysis, we identified the metabolic enzyme genes associated with the citric acid cycle that exhibited the strongest positive correlation with ZEB1. (Fig. 1A, B). This reinforces our selection of Drp1 as a target for our study, as it is one of the genes with the highest positive correlation. The crucial component of mitochondrial fusion, Mfn2, also exhibits a strong positive correlation in both HCC and CCA, further undermining ZEB1’s role in mitochondrial dynamics.

Another noteworthy observation is the strong correlation between ZEB1 and ATP citrate lyase (ACLY). ACLY, an essential metabolic enzyme that regulates fatty acid synthesis, is associated with an adverse prognosis in patients with liver malignancies, when it is upregulated^26–28^. Another correlation in the context of both primary liver cancer types involves the enzyme SUCLA2^29^. This mitochondrial matrix enzyme plays a crucial role in the reversible conversion of succinyl-CoA to succinate and acetoacetyl-CoA in the TCA cycle. Currently, there is a lack of further research in the existing literature regarding the potential involvement of SUCLA2 in primary liver malignancies, therefore providing a possible further interesting target in research.

The high correlation of ZEB1 with various key metabolic enzymes, especially enzymes involved in mitochondrial dynamics, underlines its essential role in cell metabolism, consistent with existing literature^10,17,19,30^. Further correlated genes of ZEB1 and mitochondrial metabolism are displayed in (Supplementary Fig. 1).

**Fig. 2:**
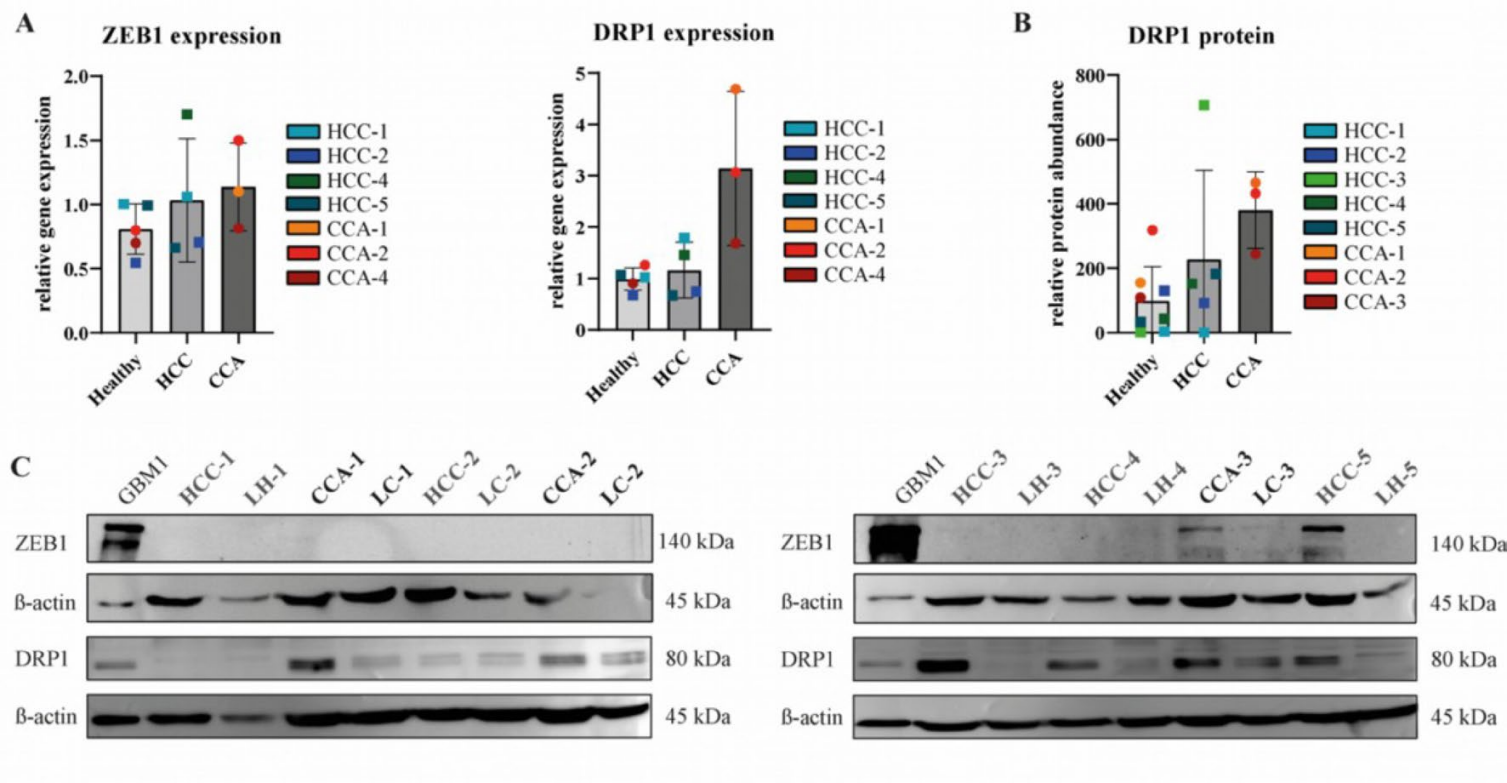
ZEB1 and Drp1 protein and RNA abundance in healthy and tumor liver tissue samples of patients. (**A**) ZEB1 and Drp1 expression on RNA levels. Normal liver tissue classified as ‘Healthy’ comprises of 5 biological replicates of HCC and CCA patients, ‘HCC’ transcriptional expression of 4 biological replicates, ‘CCA’ transcriptional expression of 3 biological replicates (**B**) Drp1 expression in patient matched healthy and tumor tissue samples on protein levels. Quantitative analysis of the corresponding Western blot of DRP1 in (**C**). Biological replicates: ‘Healthy’ *n* = 7, ‘HCC’= 5, CCA = 3 (**C**) Corresponding Western Blot results of ZEB1 and Drp1 protein abundance in healthy and tumor liver tissue. β-actin was used as housekeeping protein. ZEB1 protein abundance was observed in CCA-3 and HCC-5. The full western blot images can be obtained in the supplementary dataset.

To validate our findings from public data sets we utilized well established liver tumor cell lines cultured in monolayers. Spontaneous activation levels of ZEB1 protein and RNA expression allowed the designation of Huh7, HepG2, HuCC-T1, HuCC-A1 and TFK1 as ZEB1^low^ and Snu475, Snu387, Snu423 as ZEB1^high^ cancer cell lines (Fig. 2B). We defined a threshold of a threefold higher ZEB1 expression at the RNA level in the Zeb1^high^ cell lines. Given that monolayer cancer cell lines often exhibit limitations in the genetic stability of key tumor characteristics and can display discrepancies between mRNA and protein expression, and considering that Huh7 is well-documented as a ZEB1-independent, low-expression model we classified Huh7 as a ZEB1^low^ cell line^31,32^.

Targeted mRNA and protein quantification confirmed the positive correlation of ZEB1 activation and mitochondrial fission promoter Drp1 (Fig. 1C,D). Our ZEB1^high^ cell lines are all among the cell lines exhibiting the highest expression of Drp1 in both RNA and protein levels, excluding TFK-1, the sole exception. TFK1, an extrahepatic CCA cell lines, exhibits low ZEB1 transcriptional and protein abundance, whereas Drp1 expression was high (Fig. 1C, D). All CCA cell lines were classified as ZEB1^low^, therefore we chose to place emphasis on HCC cell lines.

We additionally performed RNA level analysis for Mfn2 (Suppl. Fig. S2A). However, our findings in the cancer cell lines did not indicate a significant upregulation of Mfn2 in ZEB1^high^ cell lines. Consequently, we proceeded with our investigation with mitochondrial fission marker Drp1.

### Screening of primary liver tumor tissues for ZEB1 and Drp1

To evaluate the expression of ZEB1 and Drp1 in our primary liver tumor and surrounding healthy liver tissue we screened our patients on both transcriptional and protein levels. A total number of 8 tumor and healthy / non tumor patient-matched samples were screened (Table 1). Due to the limited tissue availability, not all patients could be screened at the transcriptional level. We prioritized protein level analysis, as it provides more meaningful and informative data. On transcriptional levels, there was a trend towards higher levels of ZEB1 and Drp1 expression in tumor samples compared to adjacent healthy liver tissues. Increased Drp1 expression on protein levels can be observed in patients CCA-1, CCA-2, CCA-3, and HCC-1 (Fig. 2B).

Next up, we conducted our analysis on protein levels, the glioblastoma stem cell line GBM1, knowing to be highly positive for ZEB1, was used as positive control^33^. ZEB1 protein abundance was detected in 25% of liver tumors in the patient cohort (HCC-5 and CCA-3). Whereas ZEB1 was not detected in non-tumor healthy tissue, except for healthy LC-3 (Fig. 2A). In this patient ZEB1 protein abundance in tumor tissue was high in our screen. Due to the study’s design, which involves obtaining healthy liver tissue from the immediate vicinity of the tumor, the low expression of ZEB1 may be attributable to tumor cells migrating into the surrounding tissue (Fig. 2C).

To understand the nature of ZEB1 positivity in the given patients, we interrogated the available clinical data (Table 1). Patient HCC-5 emerges as notably younger (56 y) than the average age of the corresponding HCC cohort (68.2 y). Furthermore, this patient developed local tumor recurrence and lung metastasis 10 months after surgical resection, so that systemic chemotherapy with Atezolizumab and Bevacizumab was initiated. HCC-1 and HCC-2 patient developed no recurrence 3 years after surgery. HCC-3 patient developed peritoneal metastasis 3 month after surgical resection and had a stable disease under the treatment with Atezolizumab and Bevacizumab. HCC-4 patient was lost in follow up. In patient CCA-3, we considered the poor differentiation and oncological outcome to be noteworthy. The tumor demonstrates invasive growth into lymphatic vessels and veins, accompanied by infiltration in regional lymph nodes. After treatment with adjuvant systemic chemotherapy (Gemcitabine/Cisplatin/Durvalumab) the patient developed local tumor recurrence 2 years after surgery (Brachytherapy treatment). 8 months later there were multiple tumor metastases in the liver and lung. CCA-1 patient also developed tumor recurrence 18 months after surgical resection, which resected again. Until now (12 months) this patient had no signs of tumor recurrence. Furthermore, both patients with ZEB1^+^ tumors exhibit no clinically reported signs of cirrhosis, which is especially compelling in patient HCC-5 given that hepatocellular carcinoma commonly emerges in livers previously affected by damage or deterioration.

Furthermore, we observed significantly upregulated protein levels of Drp1 in tumor tissues compared to according healthy samples (Fig. 2C). These findings are consistent with our previously discussed findings in cell lines, undermining ZEB1s impact on the expression of mitochondrial fission protein Drp1. Notably, in our ZEB1 positive tissue samples CCA-3 and HCC-5 we also observed heightened Drp1 expression on protein levels. Whereas also in ZEB1 negative samples high protein levels of Drp1 were detected (CCA-1, HCC-3, HCC-4).

### Evaluation of mitochondrial metabolism: mitotracker deep red analysis, JC-1 assay for mitochondrial membrane potential, and seahorse analysis to evaluate glucose metabolism and their mitochondrial fitness

**Fig. 3:**
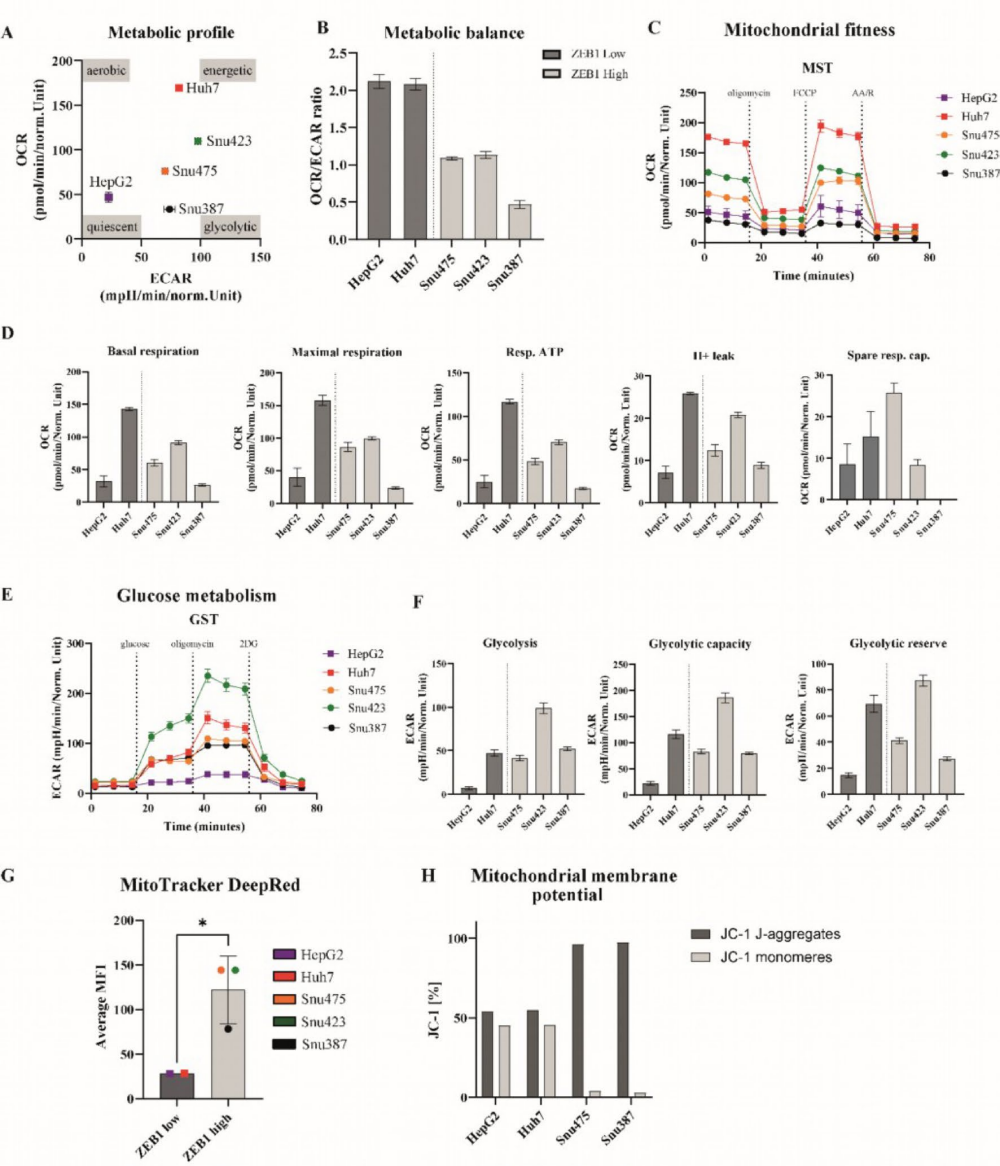
Evaluation of mitochondrial fitness and glucose metabolism in ZEB1^high^ and ZEB1^low^ liver cancer cell lines. (**A**–**C**) metabolic profile and metabolic balance for HCC cell lines, ZEB1^high^ cell lines exhibit a decreased metabolic balance compared to ZEB1^low^ cell lines. (**D**) Parameters of mitochondrial fitness, such as basal respiration, maximal respiration, resp. ATP and spare resp. cap. (**E**) Extracellular acidification rate as an indicator for glucose metabolism in all cell lines (**F**) Various parameters of glucose metabolism, such as glycolysis, glycolytic capacity and glycolytic reserve in HCC cell lines. (**G**) MitoTracker results showing mitochondrial content with significant reduction in ZEB1^Low^ cancer cell lines. Statistical analysis was performed using non-parametric Wilcoxon test, * = *p* < 0.05 (**H**) Mitochondrial membrane potential assessed in FACS analysis using JC-1.

The maintenance of mitochondrial homeostasis involves the coordinated regulation of mitochondrial biogenesis and mitophagy, considering factors such as the cells’ energy requirements. We aim to contribute valuable insights into the intricate interplay between ZEB1 and mitochondrial dynamics in primary liver cancer cells, regarding mitochondrial mass and metabolic status. To gain insight we conducted MitoTracker DeepRed (to assess mitochondrial biomass), JC-1 assay (to assess mitochondrial membrane potential) and Seahorse extracellular flux analysis (to valuate glucose metabolism and their mitochondrial fitness) with the studied HCC (Fig. 3) and CCA cell lines (Suppl. Fig. S2)- Due to the limited ZEB1 expression in CCA cell lines we are only discussing our findings in HCC cell lines. Data for mitochondrial metabolism of CCA cell lines are provided in (Supplementary Fig. 2).

The metabolic profile and the metabolic balance quickly visualize the cells energy homeostasis (Fig. 3A). Data reveals that three ZEB1^high^ cell lines are less aerobic than Huh7 and more glycolytic than HepG2. The trend can be reinforced through an assessment of the metabolic profile and metabolic balance, delineated by the ratio of OCR to ECAR. The metabolic balance serves as a metric to ascertain, whether cell lines favor oxidative or glycolytic pathways for energy consumption. All three ZEB1^high^ cell lines exhibit a relatively diminished ratio of 0.5-1, indicative of a pronounced utilization of glucose for energy metabolism, while our ZEB1^low^ cell lines exhibit ratios of around 2.0.

All ZEB1 positive cell lines exhibit high levels of glycolysis, glycolytic capacity and glycolytic reserve (Fig. 3B). Huh7 shows the second-highest glycolytic activity, however, as the most metabolically active cell line in our portfolio it is also exhibiting higher levels of mitochondrial metabolism.

We discovered that mitochondrial activity is diminished in all parameters, such as basal respiration and respiratory ATP, within our ZEB1^high^ cell lines compared to Huh7 (Fig. 3C). Furthermore, basal respiration and maximum respiration of the ZEB1^low^ cell lines HepG2 exhibit a comparable level of mitochondrial activity to the ZEB1^high^ cell lines (Fig. 3C).Finally, we assessed the total mitochondrial content in our cells using MitoTracker. I The MitoTracker Deep red signal was significantly increased in our ZEB1^high^ cancer cell lines, which is associated with larger mitochondrial biomass (Fig. 3G). JC-1 assay of ZEB1^High^ cell lines displays a high number of JC-1-aggregates indicating a high mitochondrial membrane potential, whereas ZEB1^Low^ cell lines show a lower membrane potential with almost balanced JC1-aggregates and monomers. These data indicate structurally intact mitochondria in ZEB1^High^ cell lines.

In summary, the results described above indicate that our ZEB1^high^ cell lines exhibit a large amount of functional mitochondria, while energy production mainly depends on glucose metabolism.

### Treatment with small molecule mitochondrial division inhibitor Mdivi-1 effectively causes apoptosis in HCC cells independent of ZEB1 status

Next, to test the hypothesis if the ZEB1 associated metabolic signature in liver cancer cells opens opportunity for therapeutic intervention. We chose Mdivi-1, a widely studied pharmacological agent to inhibit Drp1 activity.

**Fig. 4:**
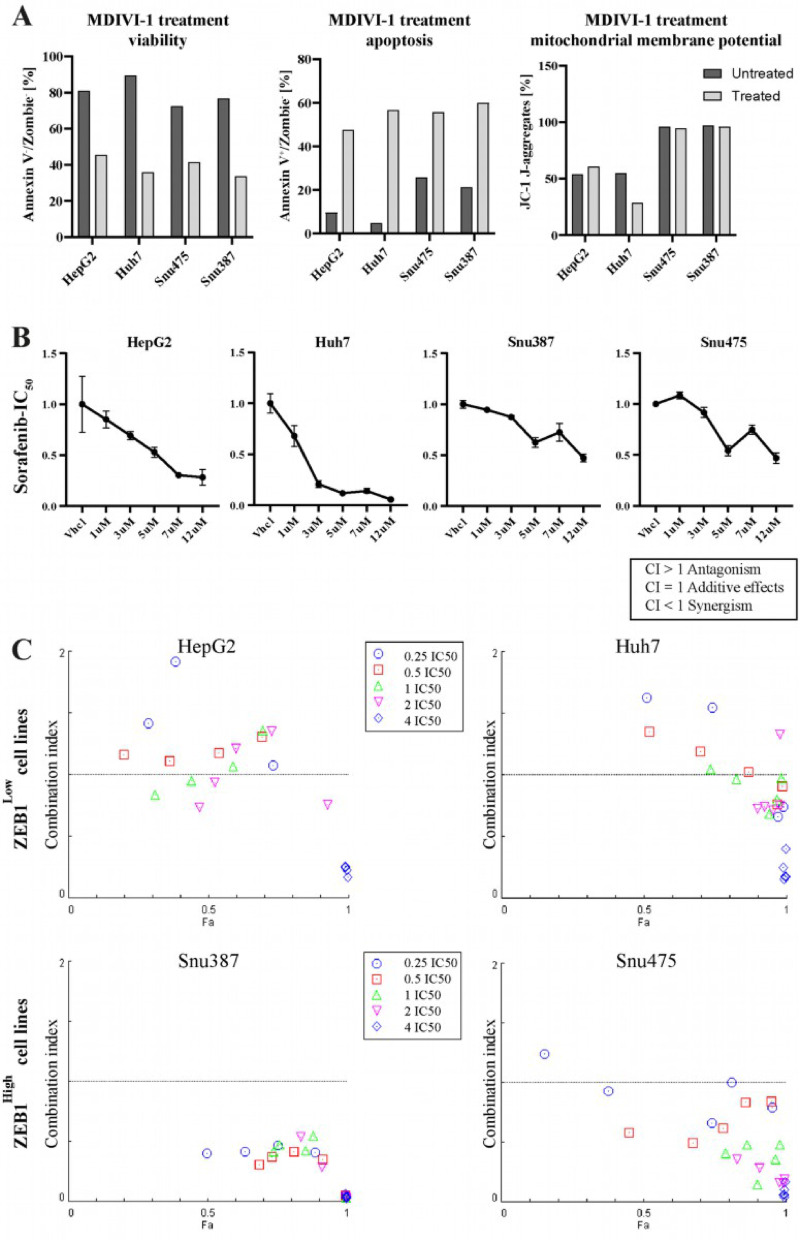
Apoptosis and mitochondrial membrane potential in MDIVI-1 treated cells and Combination Index plots for cells treated with a combination treatment of MDIVI-1 and Sorafenib. (**A**) Cell viability and apoptosis levels in cell lines after 48 h of treatment with MDIVI-1, assessed in FACS analysis. JC-1 aggregate levels indicating mitochondrial membrane potential after MDIVI-1 treatment, assessed in FACS analysis (**B**) Sorafenib titration results to determine the IC50 used in our combination treatment. Cell viability after 72 h measured by fluorescence is displayed. (**C**) Combination index < 1 indicates synergism, > 1 indicates antagonism, CI = 1 indicates additive effects. Our ZEB1^high^ cell lines exhibit a stronger synergism, also in lower drug concentrations compared to ZEB1^low^ cell lines with only additive or at lower levels antagonistic effects. Each data point represents the effect of a specific concentration of Sorafenib in combination with the indicated concentration of MDIVI-1 and is based on two technical replicates.

First, we examined whether MDIVI-1 itself induces apoptosis in HCC cell lines revealing a severe induction of apoptosis in all models (Fig. 4A). Additionally, mitochondrial membrane potential was examined using JC-1 assay, providing preserved mitochondrial membrane potential in all cell lines after MDIVI-treatment except for Huh7.

### Treatment with Mdivi-1 potentiates growth Inhibition of Sorafenib in ZEB1^high^ HCC cells

Next, we analyzed a combination therapy of MDIVI-1 with protein kinase inhibitor Sorafenib. IC50 concentrations from the literature were screened for all used cell lines and the final IC50 concentration was experimentally validated (Fig. 4B). IC50s for our ZEB1^high^ cell lines were higher (10 µM) than for ZEB1^low^ Huh7 (4 µM) and HepG2 (6 µM), displaying a higher chemoresistance in our ZEB1^high^ cell lines. A higher concentration of Sorafenib also presents several challenges, including side effects, highlighting the need for combination therapies to reduce the required doses. Focusing on quantifying cellular growth, we looked for any antagonistic, additive or synergistic effects using the combination index plot (Fig. 4C). Strikingly, in ZEB1^high^ tumor cells the combination treatment showed stronger reduction in tumor viability compared to mono-drug treatment. This was also observed in lower IC50 concentrations in the combination treatment, displaying strongly synergistic effects of Mdivi-1 and Sorafenib especially at lower doses. A synergistic effect can be advantageous in reducing chemotherapy dosage and consequently minimizing adverse side effects. In ZEB1^low^ cancer cells this striking anti-tumorigenic potential of the combination-drug treatment was not observed, showing synergism at fewer dosage combinations and even antagonism in some cases (Fig. 4C).

## Discussion

Our work strongly enforces the need to establish clinical programs for testing the effect of pharmacological-mediated blockage of mitochondrial division for the treatment of hepatocellular cancer. By using elevated dosages of up to 60 µM Mdivi-1, we find robust induction of apoptosis in all tested cancer models. To our knowledge, this is the first medium scale assessment of such under normoxic conditions^34–36^. Atezolizumab and Bevacizumab are the current first-line systemic therapy options of HCC. Sorafenib continues to be a crucial treatment approach, particularly in the advanced stages of HCC. We performed combination therapy tests and identified a severe synergistic treatment effect on Sorafenib resistant ZEB1^high^ HCC cell. To our knowledge, this is the first proven evidence that pharmacological strategy targeting mitochondrial division can increase efficacy of standard of care chemotherapeutics to impair HCC cell growth, thereby indicating a particular relevant clinical translational finding. We acknowledge that the results of the in vitro combination treatment trials also show antagonistic effects especially when using low concentrations in ZEB1^Low^ cell lines. A plausible explanation for the antagonistic effects observed in HepG2 cells is the relatively low mitochondrial mass inherent to this cell line, which was also demonstrated in our study. As Sorafenib has been reported to induce mitophagy and inhibit components of the respiratory chain, cells with diminished mitochondrial content may require higher concentrations of treatment to elicit a measurable response^37^. This interpretation is further supported by the finding that antagonism was most pronounced at lower drug concentrations, indicating that limited mitochondrial abundance may restrict the effectiveness of the combination therapy under these conditions.

The presented data adds to our knowledge that the fundamental cellular process of EMT is associated to metabolic reprogramming. We show that higher ZEB1 activation correlates with higher mitochondrial fission as marked with Drp1 upregulation, both in cell models and subset of patient cancer tissue samples. Additionally, examination using MitoTracker and JC-1 assay indicated an increased mitochondrial mass and high mitochondrial membrane potential within ZEB1^high^ cell lines. Nonetheless, our assessment of extracellular flux revealed diminished mitochondrial metabolism, especially oxidative phosphorylation, in these cell lines, suggesting impaired mitochondrial function. The relation of Drp1 and ZEB1 was additionally explored in a combination treatment of Drp1-inhibitor Mdivi-1 and Sorafenib. Synergistic effects of the combination drug treatment were observed exclusively in ZEB1^high^ cell lines, even at lower drug concentrations. ZEB1 is a known key player in EMT activation and as a valid marker for cancer stem cells^7^. In concordance, ZEB1 is known as biomarker to predict poorer oncological outcome and metastasis risk in HCC^9,38,39^. The presented data shows evidence that Mdivi-1 may help to impair the EMT/ cancer stem cell phenotype in HCC therapy inducing sensitivity to classical treatment regimes. Further analysis of Midivi-1 treated cells, such as their clonogenic behavior in vitro are needed to verify this hypothesis.

Probing ZEB1 protein abundance in tumor cells-free liver and patient-matching tumor tissue, we observed its relevance as a tumor marker in 25% of patients only. Our data is in concordance with others, that in ZEB1 positive tumors patients suffer from poorer clinical outcome and ZEB1 is not active in healthy liver tissue^38,39^. This is in discrepancy with previous reports showing ZEB1 ‘positive’ values varying between 20% and 60% of cases^40,41^. The difference may be explained as our patients are from Western Europe and mostly Caucasian ethnicity, whereas other reposts include Asian population. Drp1 protein is activated in 6 out of 8 patients, with Drp1 levels always upregulated in tumor. This data underlines our assumption that mitochondrial fission is enhanced in tumors^42^. The strong activation of Drp1, leading to enhanced mitochondrial fission, has been frequently discussed in the context of HCC, where it promotes *de novo* fatty acid synthesis in liver cancer cells, suggesting a potential target for therapeutic intervention^43,44^. Zhan et al. further demonstrated that Drp1-mediated mitochondrial fission can contribute to cell proliferation, highlighting its significance in tumor progression^45^. The in here revealed correlation of Drp1 activity and ZEB1 activation may be an additional mechanistic explanation of previously described ZEB1s pro-proliferative role in HCC and as independent biomarker for HCC metastasis^11^.

The presented in detail functional metabolic profiling of in vitro models may help the field to depicture a reference booklet for metabolic status of those widely used classical HCC cells. We identified that ZEB1^high^/DRP1^high^ cancer cell lines have a greater mitochondrial mass with high membrane potential, indicating structurally intact mitochondria. However, functional analysis of mitochondrial metabolism shows that energy production mostly depends on glycolysis, a common property of high aggressive cancers. High ZEB1 levels support this hypothesis and possibly present a strategic adaptation of cancer cells to enhance resistance to oxidative stress even if it compromises overall mitochondrial efficiency^46^. An imbalance in mitochondrial fission and fusion activities can cause mitochondrial dysfunction, but mitochondrial formation and degradation remain highly dynamic processes regulated by diverse pathways^35^. Our findings show that the HepG2 cell line (ZEB1^low^) has mitochondrial activity comparable to the ZEB1^high^ group; however, this activity must be interpreted in the context of HepG2 relatively quiescent metabolic status. We found that MDIVI-1 efficiently induces apoptosis in all HCC cell lines, whereas the mitochondrial membrane potential is only disrupted in Huh7 cells, introduced as model with possible high flexibility of metabolic shifting between glycolysis and respiration. This indicates that the MDIVI-1 inhibition of Complex I of the respiratory chain does not provoke apoptosis in cancer cells by mitochondrial membrane potential disruption. Investigation of alternative mechanisms as increased proton leak, activation of ATP-sensitive potassium channels, and structural resilience has to be considered in future studies^47^.

Further studies are needed to clarify the synergistic effects of Mdivi-1 and Sorafenib, ideally using more clinically relevant models such as patient-derived organoids and in vivo validation in PDX models. Establishing a direct mechanistic link between ZEB1 and Drp1 will also require functional studies using ZEB1 overexpression and knockdown systems. A key limitation of this study is the relatively small patient cohort, with only 25% exhibiting ZEB1 positivity, which may limit the generalizability of our findings. Validation in larger, independent cohorts will be essential to confirm these results and to better evaluate the clinical relevance of ZEB1 as a predictive biomarker in HCC.

## Supplementary Information

Below is the link to the electronic supplementary material.

Supplementary Material 1

Supplementary Material 2

## Data Availability

Data is provided within the manuscript. Further information can be received by the corresponding author upon reasonable request.

## Abbreviations

ZEB1: Factor zinc finger E-box binding homeobox 1
Mdivi-1: Mitochondrial division inhibitor 1
EMT: Epithelial mesenchymal transition
Drp1: Dynamin-related protein 1
HCC: Hepatocellular carcinoma
CCA: Cholangiocellular adenocarcinoma
Mfn2: Mitofusin-2
BME: Basement membrane extract
MFI: Mean fluorescence intensity
OCR: Oxygen consumption rate
ECAR: Extracellular acidification rate
TCGA: The cancer genome atlas
