# Supplementary material for: ZEB1 stratifies the response to Sorafenib and Mdivi-1 combination therapy in hepatocellular carcinoma
Source: Sci Rep. 2025 Aug 19;15:30451. doi: 10.1038/s41598-025-16379-6 (PMC12365315; doi:10.1038/s41598-025-16379-6)

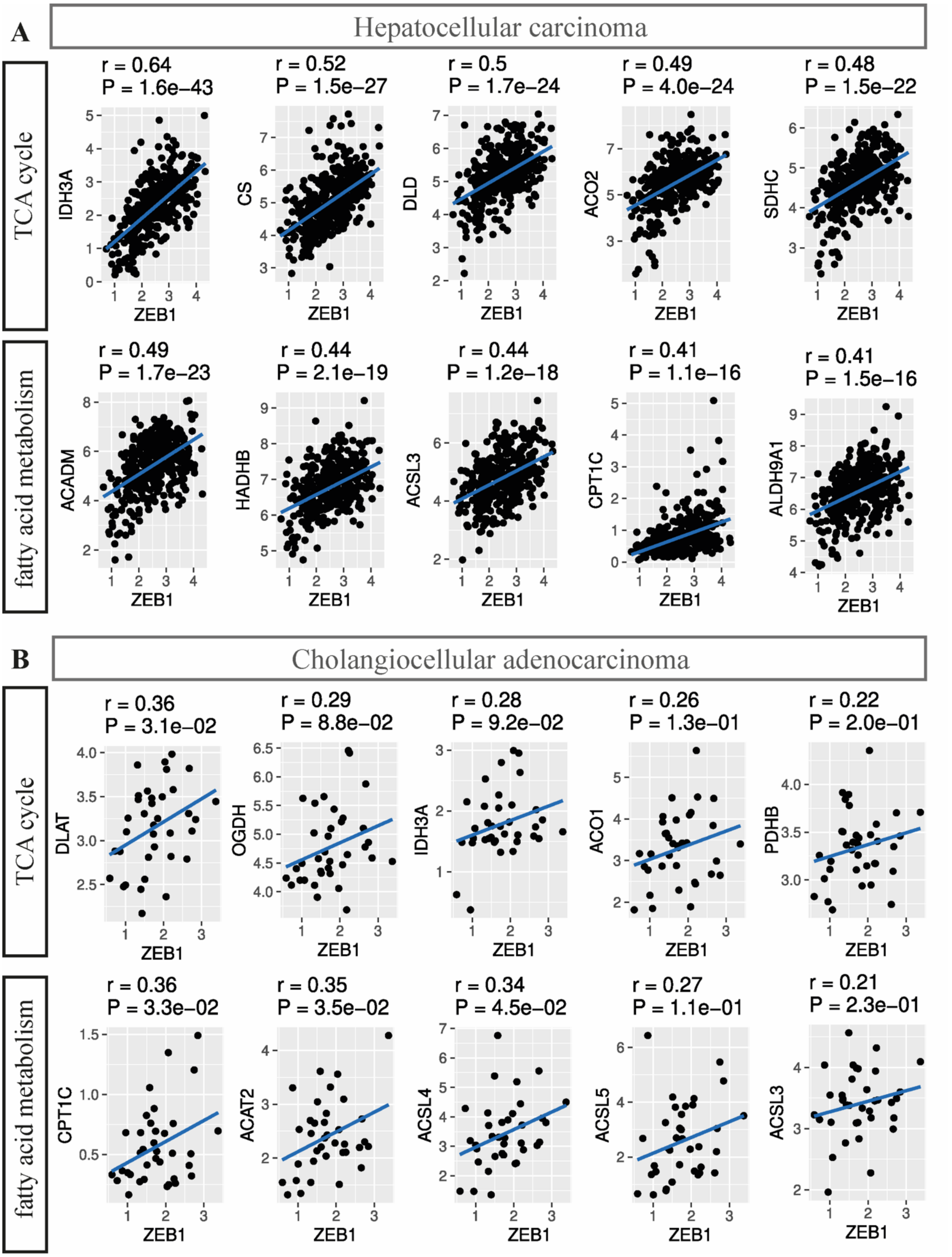

Figure S1: Bioinformatic analysis for various genes in mitochondrial metabolism correlated to ZEB1 expression on transcriptomic levels. A: Top 5 positively correlated genes in HCC in Tricarboxylic Acid Cycle (TCA Cycle) and fatty acid metabolism, Isocitrate Dehydrogenase 3 (IDH3A), Citrat synthase (CS), Dihyrdolipoamide dehydrogenase (DLD), Aconitase 2 (ACO2), Succinate Dehydrogenase complex subunit C (SDHC), Acyl-CoA Dehydrogenase Medium Chain (ACADM), Hydroxyacyl-CoA Dehydrogenase (HADHB), Acyl-CoA Synthetase long-chain family member 3 (ACSL3), Carnitine Palmitoyltransferase 1

(CPT1), Aldehyde Dehydrogenase 9 family member A1 (ALDH9A1) **B:** Top 5 positively correlated targets in CCA: Dihydrolipoyl-Transacetylase (DLAT), Alpha-ketoglutarate Dehydrogenase (OGDH), Aconitase 1 (ACO1), Pyruvat Dehydrogenase beta (PDHB), Acetyl-CoA Acetyltransferase (ACAT2), Long-chain-fatty-acid—CoA ligase 4 (ACSL4), Long-chain-fatty-acid—CoA ligase 5 (ACSL3)

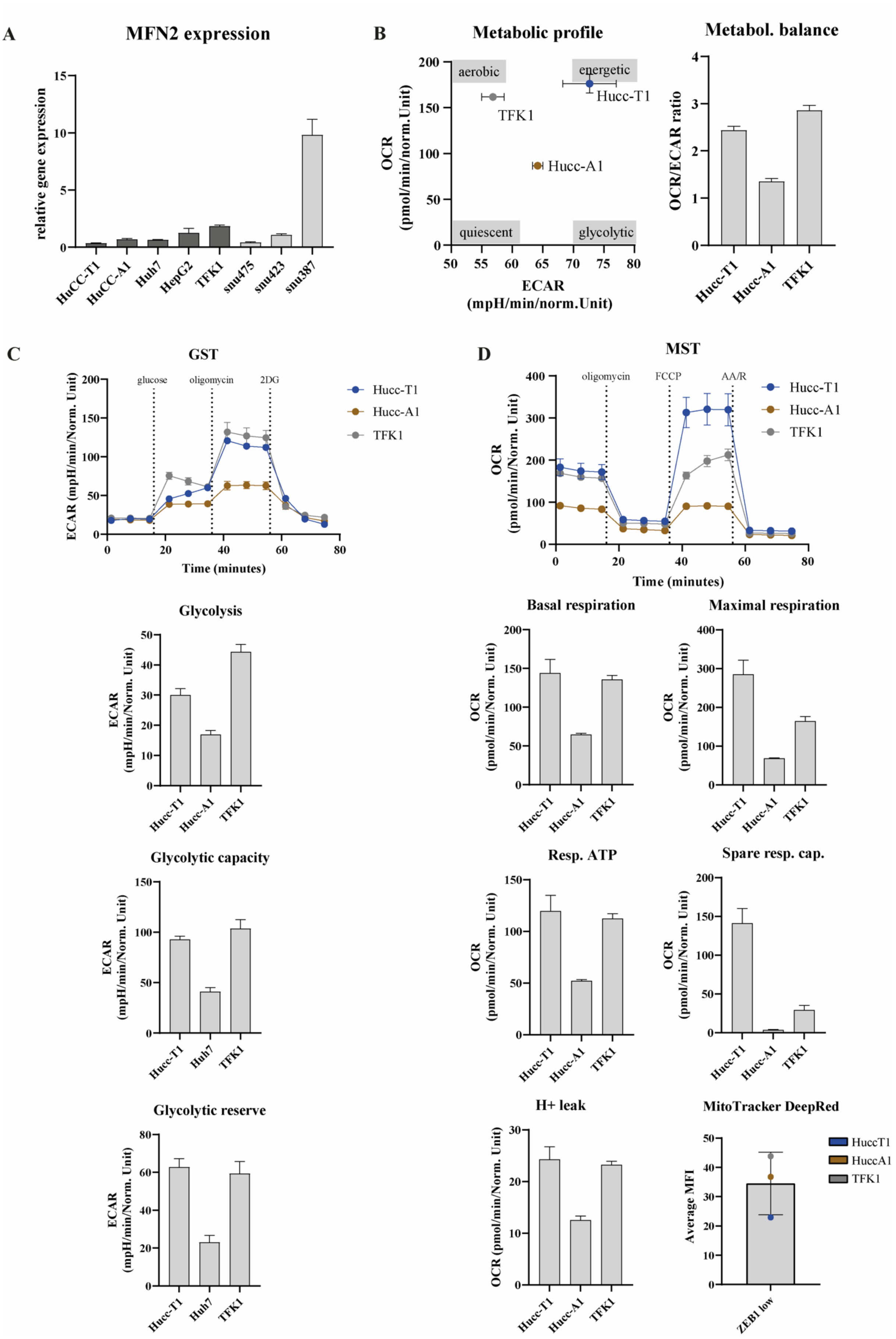

A
MFN2 expression
relative gene expression
HuCC-T1
HuCC-A1
Huh7
HepG2
TFK1
snu475
snu423
snu387
B
Metabolic profile
aerobic
energetic
quiescent
glycolytic
TFK1
Hucc-T1
Hucc-A1
OCR (pmol/min/norm.Unit)
ECAR (mpH/min/norm.Unit)
Metabol. balance
OCR/ECAR ratio
C
GST
glucose
oligomycin
2DG
ECAR (mpH/min/Norm. Unit)
Time (minutes)
D
MST
oligomycin
FCCP
AA/R
OCR (pmol/min/Norm. Unit)
Time (minutes)
Hucc-T1
Hucc-A1
TFK1
Glycolysis
Glycolytic capacity
Glycolytic reserve
ECAR (mpH/min/Norm. Unit)
Basal respiration
Maximal respiration
Resp. ATP
Spare resp. cap.
H+ leak
MitoTracker DeepRed
Average MFI
ZEB1 low
HuccT1
HuccA1
TFK1

Supplementary Figure 2: Evaluation of mitochondrial fitness and glucose metabolism in ZEB1^{high} and ZEB1^{low} CCA cell lines. A: Mfn2 expression on transcriptomic levels in the cell lines used in our study **B:** metabolic profile and metabolic balance for HCC cell lines, ZEB1^{high} cell lines exhibit a decreased metabolic balance compared to ZEB1^{low} cell lines. **C:** Various parameters of glucose metabolism, such as glycolysis, glycolytic capacity and glycolytic reserve in CCA cell lines. **D:** Parameters of mitochondrial fitness, such as basal respiration, maximal respiration, resp. ATP and spare resp. cap.

Supplementary figure 3: Bioinformatic data sets

A: Correlation of ZEB1 with relevant enzymes of fatty acid synthesis, bile acid synthesis and citrate cycle in HCC

B: Correlation of ZEB1 with relevant enzymes of fatty acid synthesis, bile acid synthesis and citrate cycle in CCC

A:

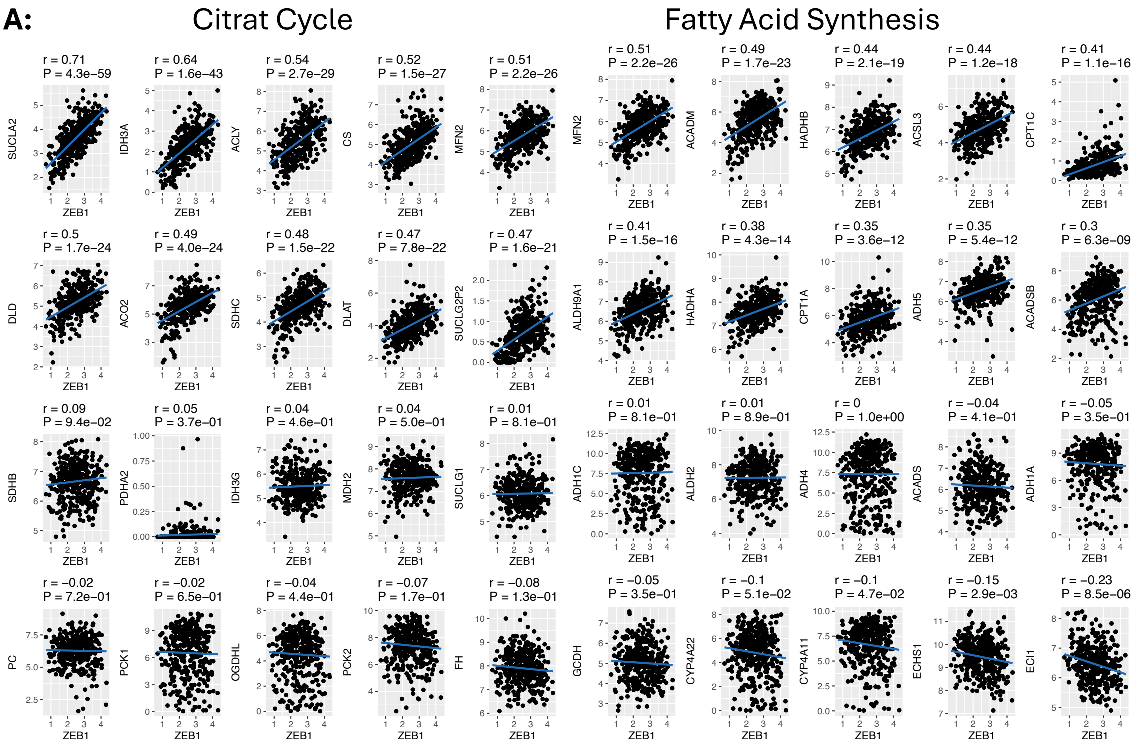

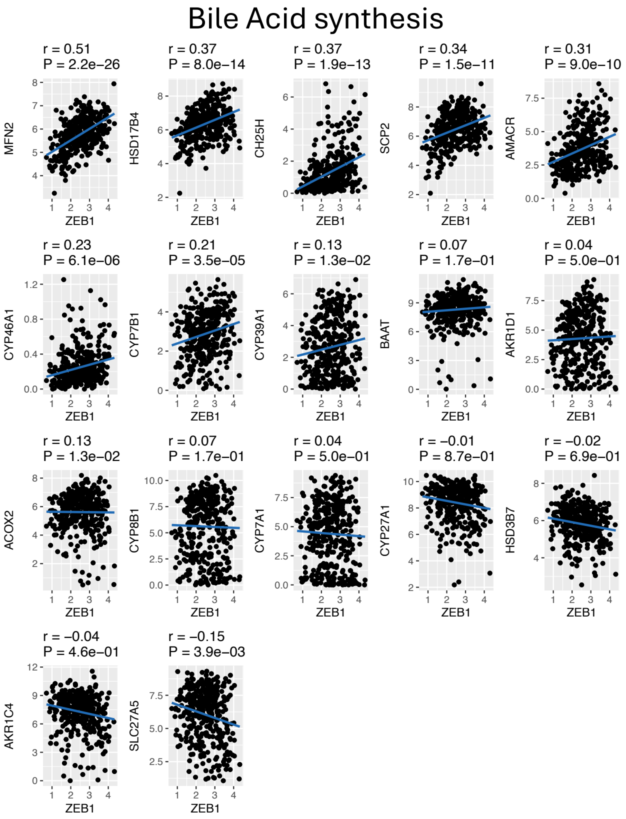
Bile Acid synthesis
r = 0.51
P = 2.2e−26
MFN2
r = 0.37
P = 8.0e−14
HSD17B4
r = 0.37
P = 1.9e−13
CH25H
r = 0.34
P = 1.5e−11
SCP2
r = 0.31
P = 9.0e−10
AMACR
r = 0.23
P = 6.1e−06
CYP46A1
r = 0.21
P = 3.5e−05
CYP7B1
r = 0.13
P = 1.3e−02
CYP39A1
r = 0.07
P = 1.7e−01
BAAT
r = 0.04
P = 5.0e−01
AKR1D1
r = 0.13
P = 1.3e−02
ACOX2
r = 0.07
P = 1.7e−01
CYP8B1
r = 0.04
P = 5.0e−01
CYP7A1
r = −0.01
P = 8.7e−01
CYP27A1
r = −0.02
P = 6.9e−01
HSD3B7
r = −0.04
P = 4.6e−01
AKR1C4
r = −0.15
P = 3.9e−03
SLC27A5
ZEB1

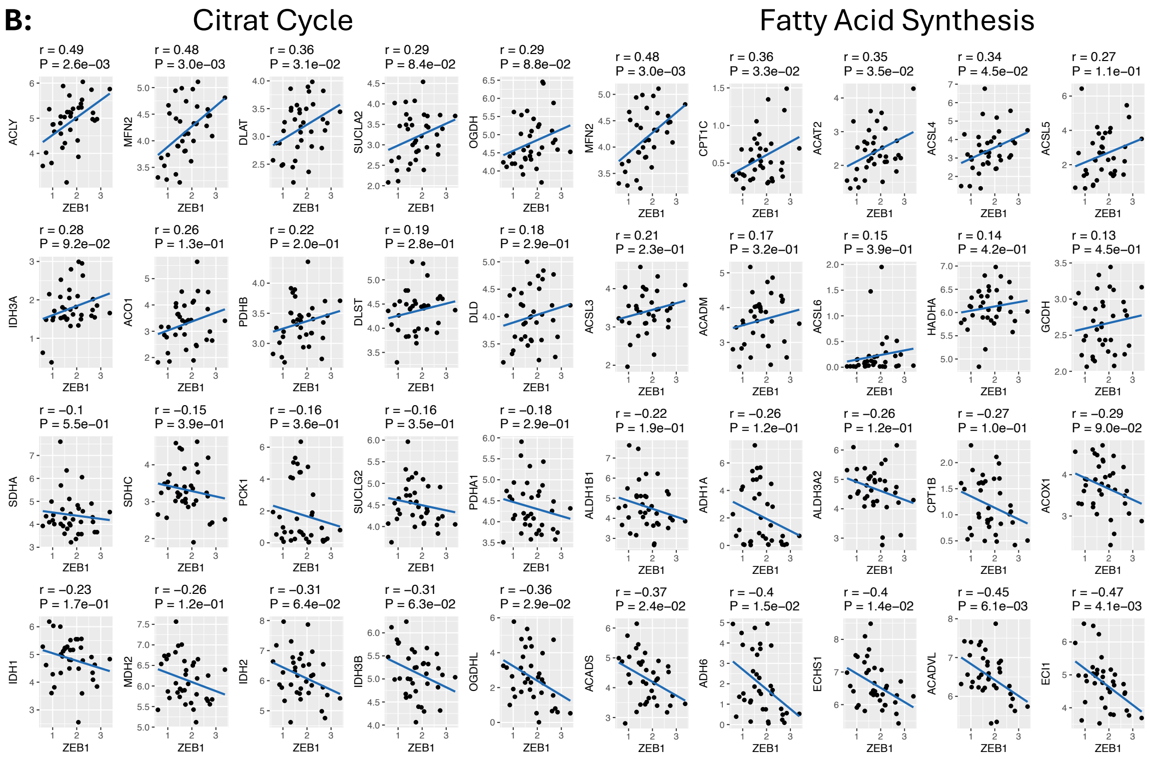
B:
Citrat Cycle
Fatty Acid Synthesis
r = 0.49
P = 2.6e−03
ACLY
r = 0.48
P = 3.0e−03
MFN2
r = 0.36
P = 3.1e−02
DLAT
r = 0.29
P = 8.4e−02
SUCLA2
r = 0.29
P = 8.8e−02
OGDH
r = 0.48
P = 3.0e−03
MFN2
r = 0.36
P = 3.3e−02
CPT1C
r = 0.35
P = 3.5e−02
ACAT2
r = 0.34
P = 4.5e−02
ACSL4
r = 0.27
P = 1.1e−01
ACSL5
r = 0.28
P = 9.2e−02
IDH3A
r = 0.26
P = 1.3e−01
ACO1
r = 0.22
P = 2.0e−01
PDHB
r = 0.19
P = 2.8e−01
DLST
r = 0.18
P = 2.9e−01
DLD
r = 0.21
P = 2.3e−01
ACSL3
r = 0.17
P = 3.2e−01
ACADM
r = 0.15
P = 3.9e−01
ACSL6
r = 0.14
P = 4.2e−01
HADHA
r = 0.13
P = 4.5e−01
GCDH
r = −0.1
P = 5.5e−01
SDHA
r = −0.15
P = 3.9e−01
SDHC
r = −0.16
P = 3.6e−01
PCK1
r = −0.16
P = 3.5e−01
SUCLG2
r = −0.18
P = 2.9e−01
PDHA1
r = −0.22
P = 1.9e−01
ALDH1B1
r = −0.26
P = 1.2e−01
ADH1A
r = −0.26
P = 1.2e−01
ALDH3A2
r = −0.27
P = 1.0e−01
CPT1B
r = −0.29
P = 9.0e−02
ACOX1
r = −0.23
P = 1.7e−01
IDH1
r = −0.26
P = 1.2e−01
MDH2
r = −0.31
P = 6.4e−02
IDH2
r = −0.31
P = 6.3e−02
IDH3B
r = −0.36
P = 2.9e−02
OGDHL
r = −0.37
P = 2.4e−02
ACADS
r = −0.4
P = 1.5e−02
ADH6
r = −0.4
P = 1.4e−02
ECHS1
r = −0.45
P = 6.1e−03
ACADVL
r = −0.47
P = 4.1e−03
ECI1
ZEB1

Bile Acid Synthesis

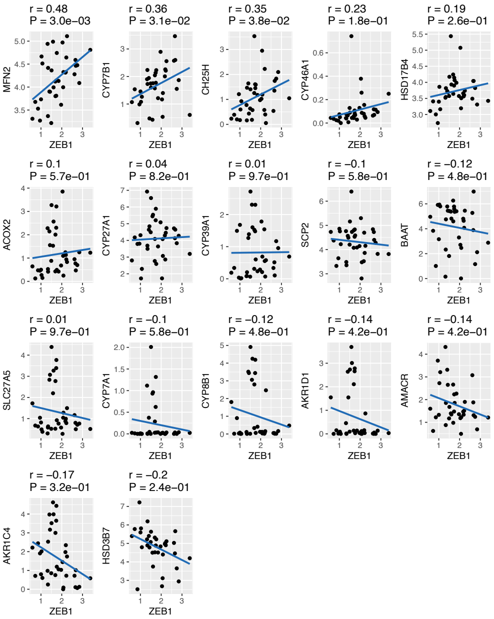

Figure 1C: ZEB1

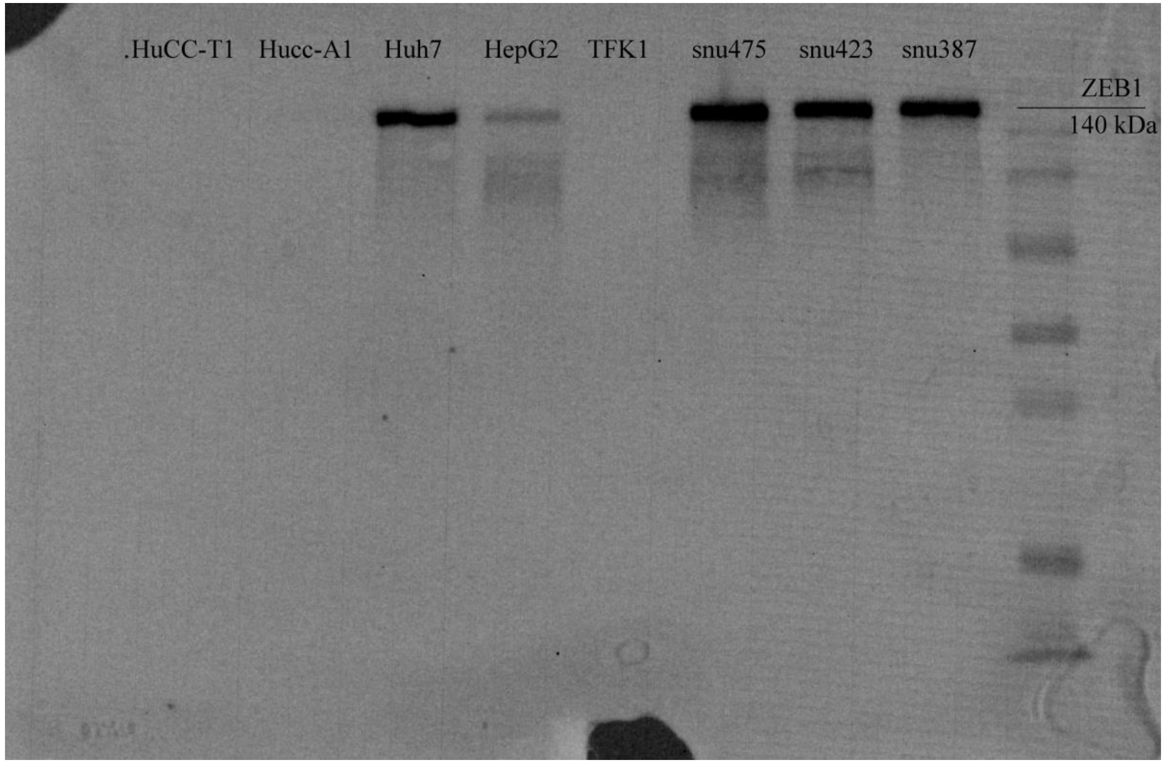

Figure 1C: actin

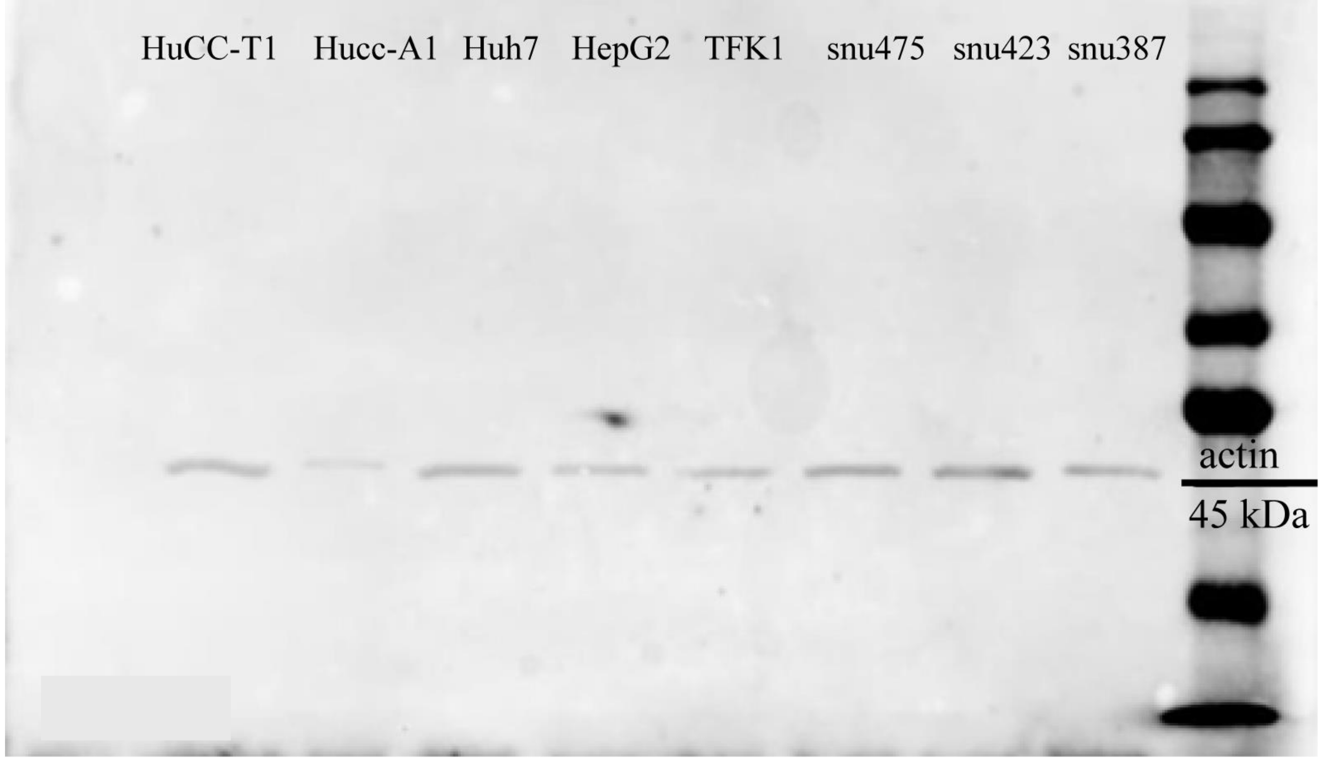

Figure1C: DRP1

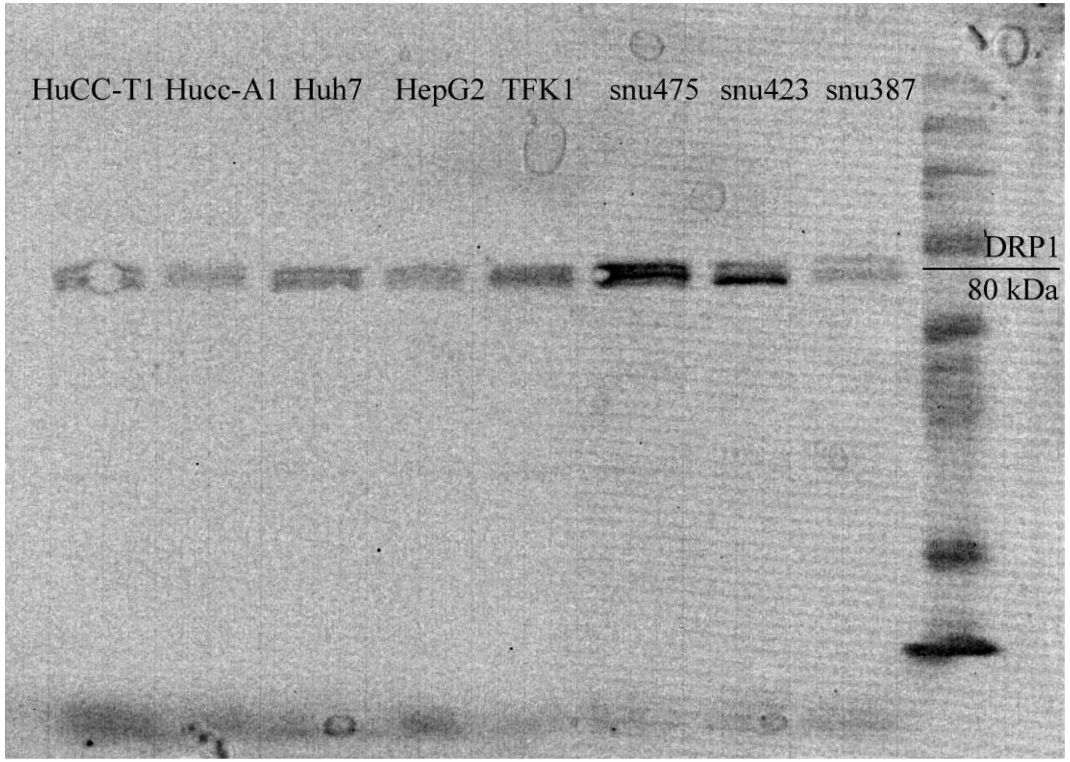

Figure1C: actin

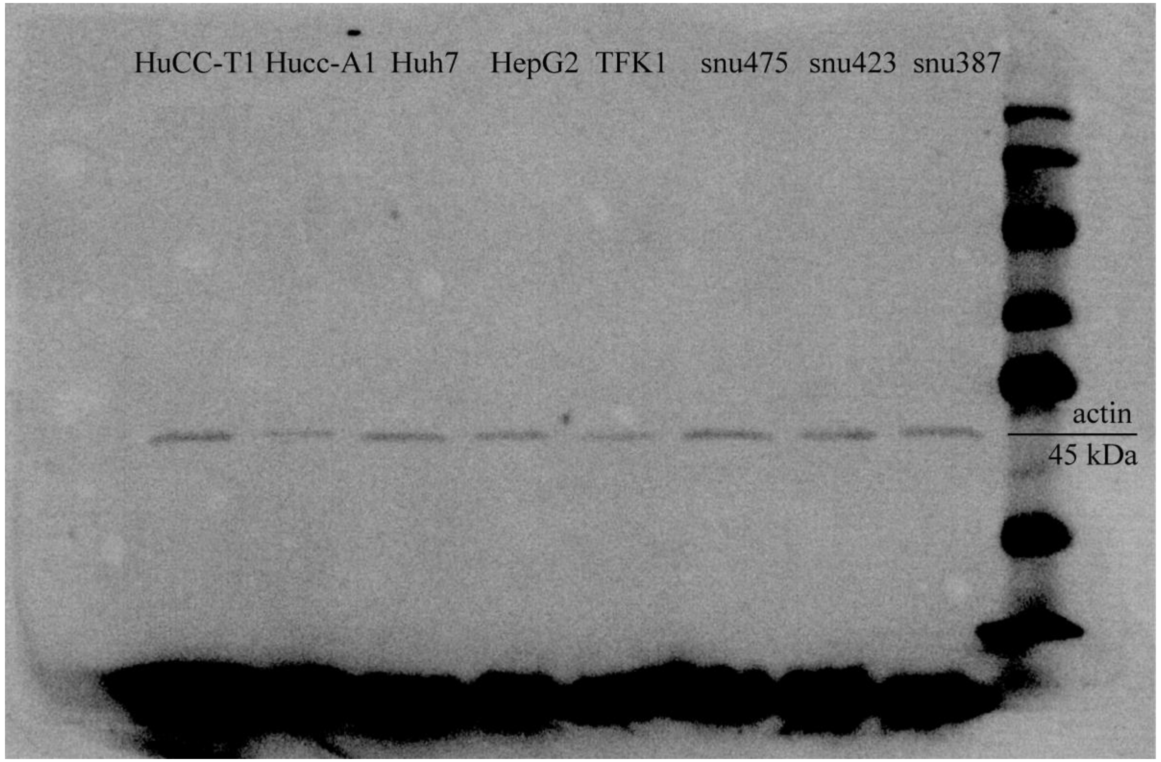

Figure 2C: ZEB1

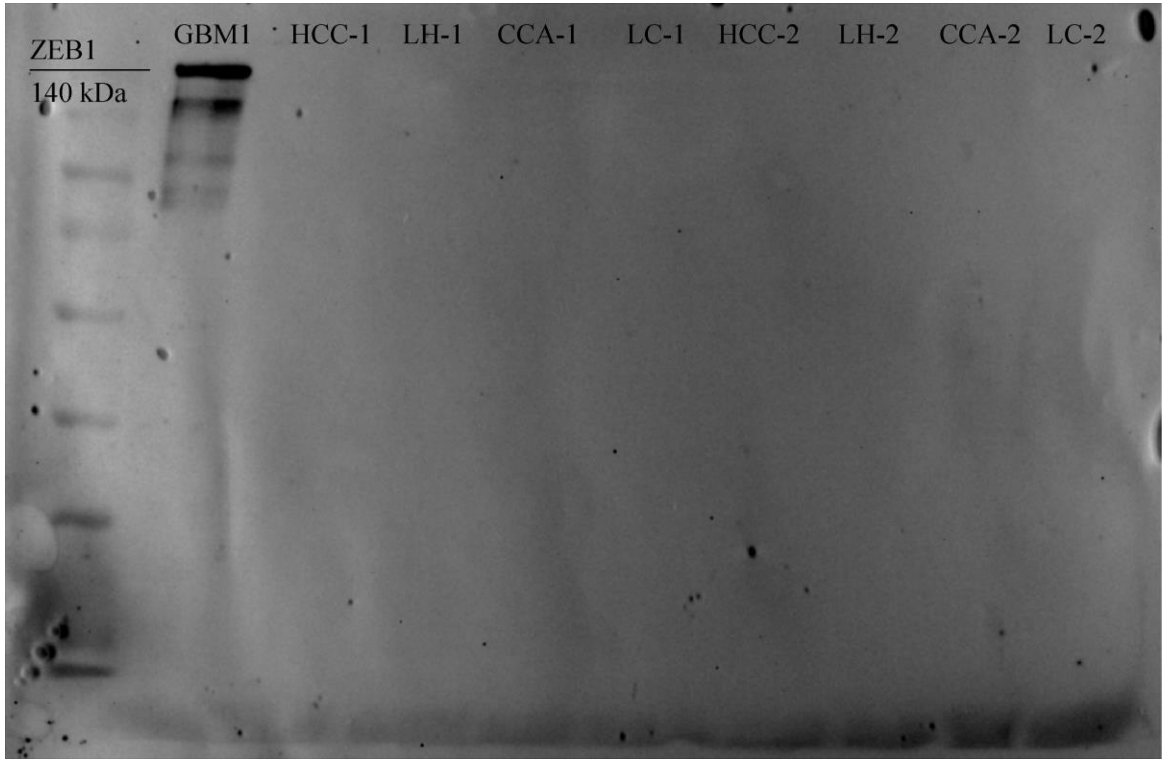

Figure 2C: actin

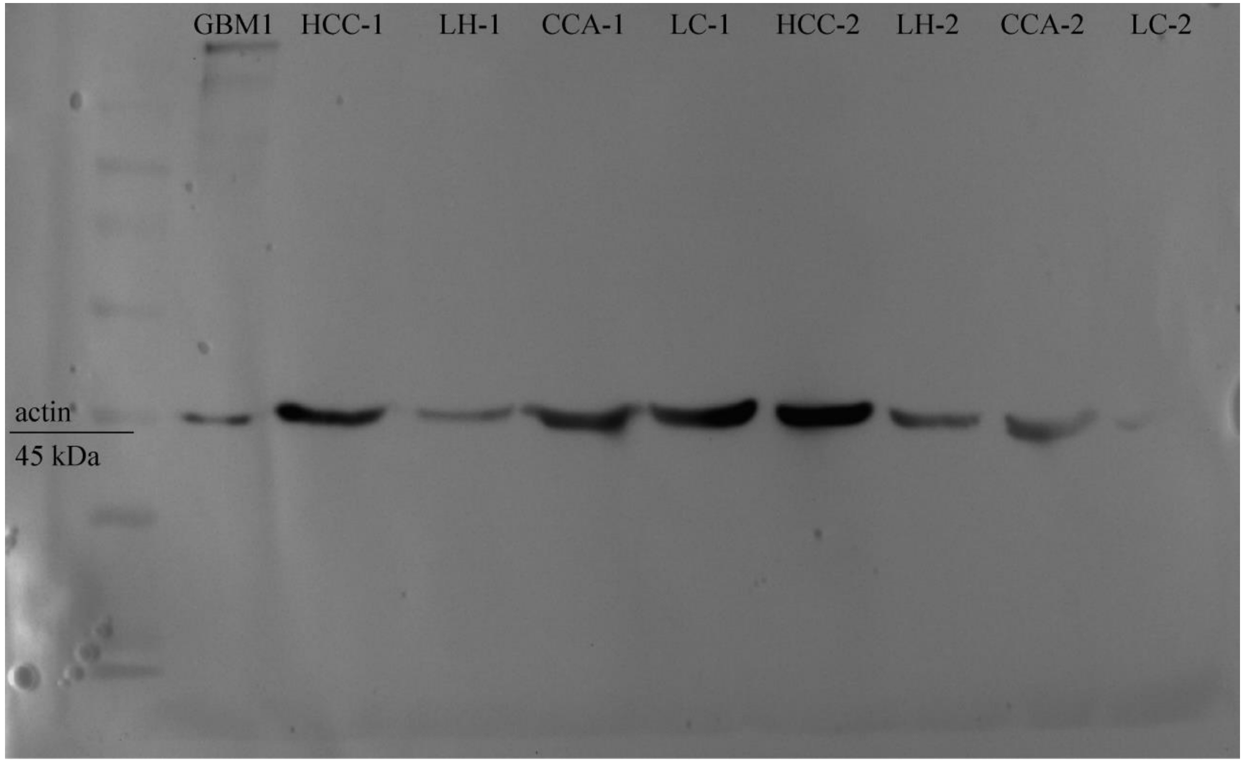

Figure 2C: DRP1

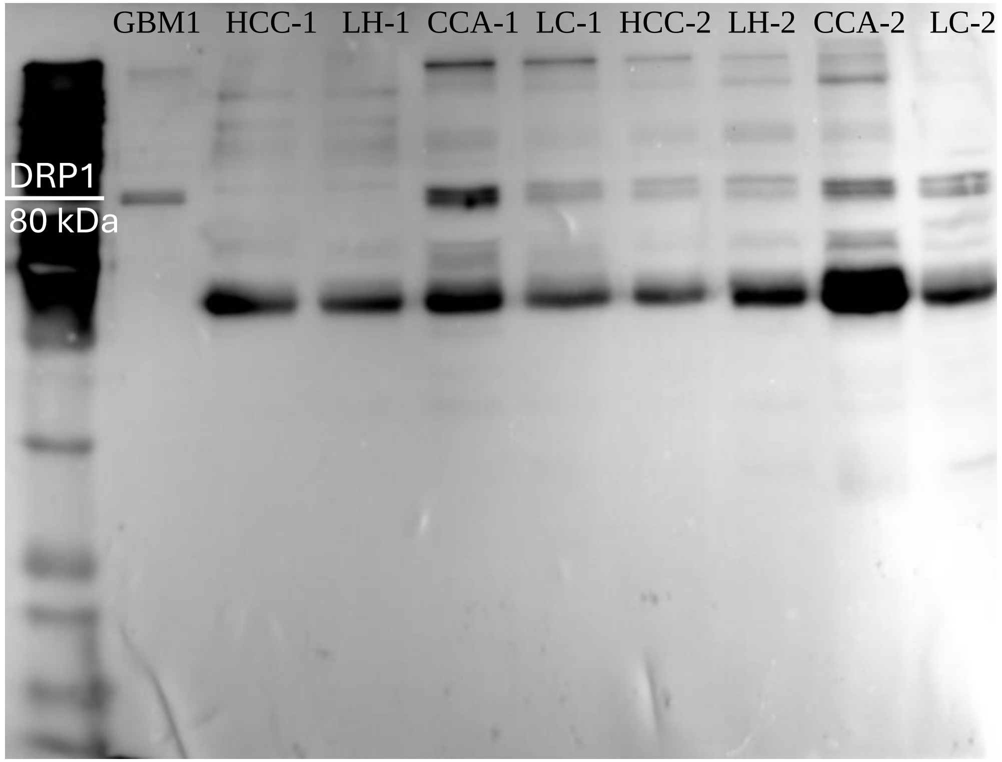

Figure 2C: actin

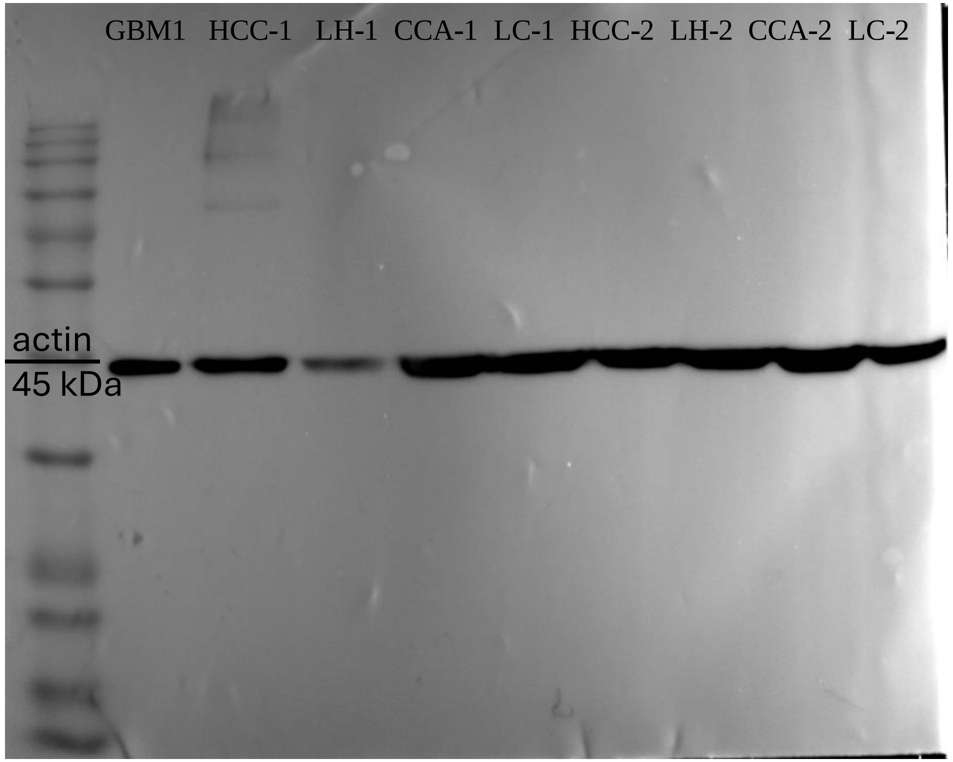

Figure 2C: ZEB1

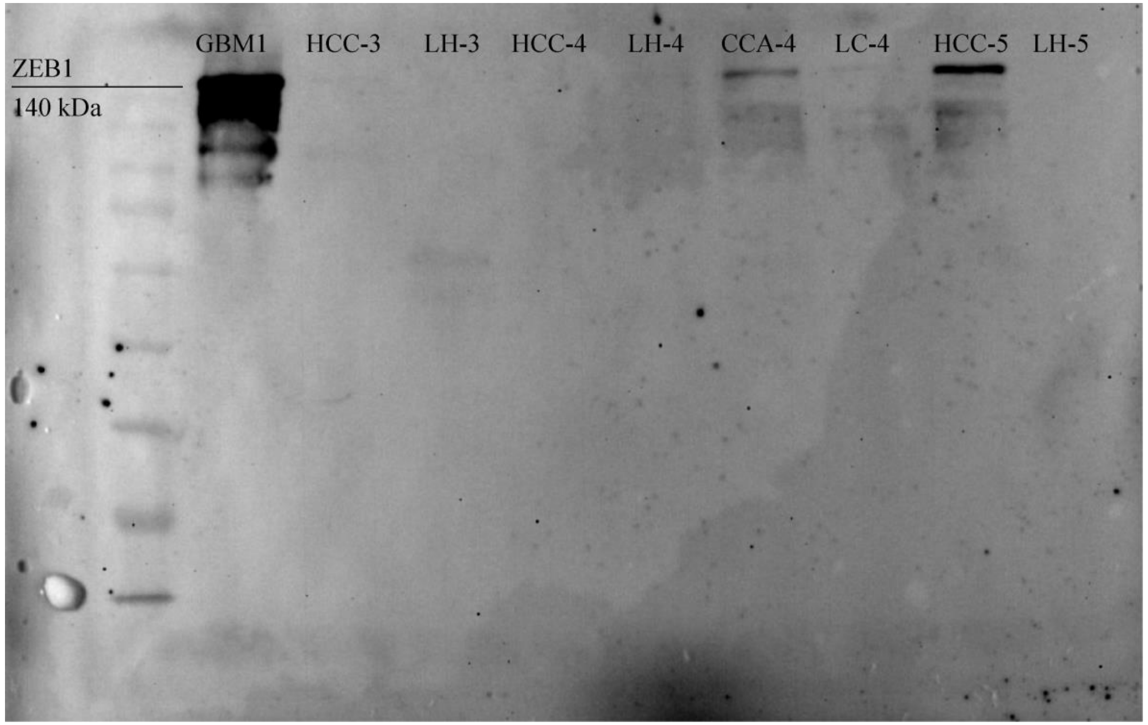

Figure 2C: actin

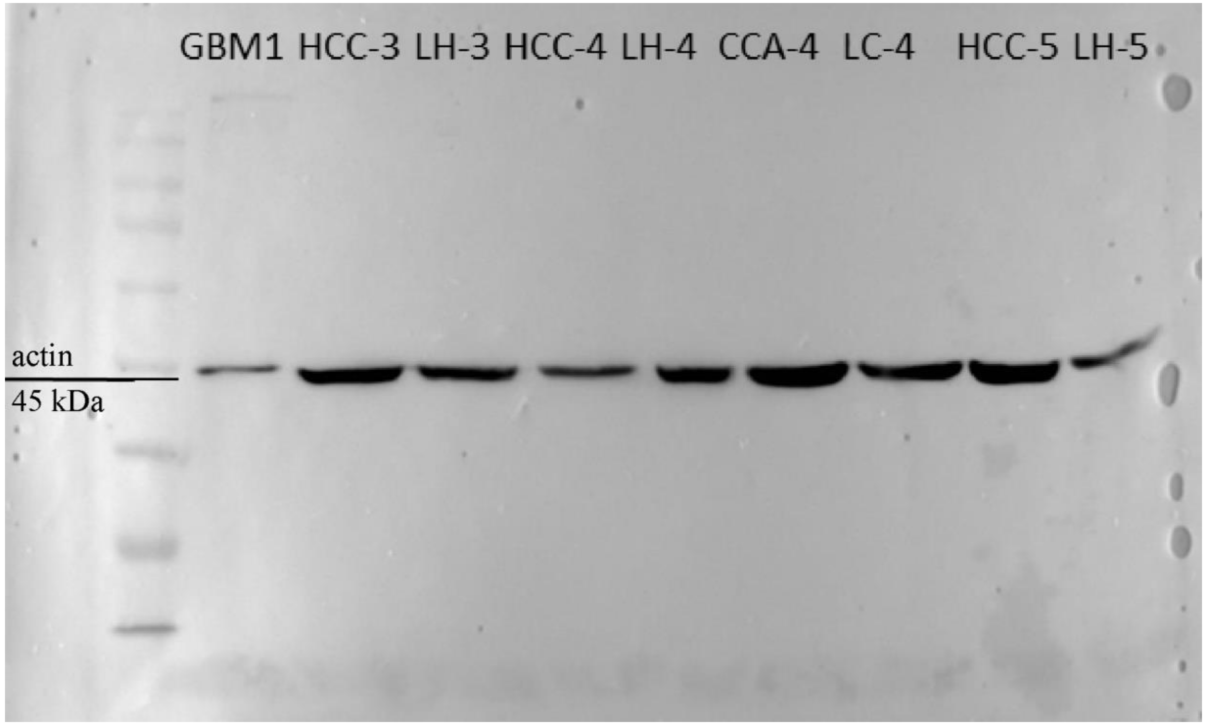

Figure 2C: DRP1

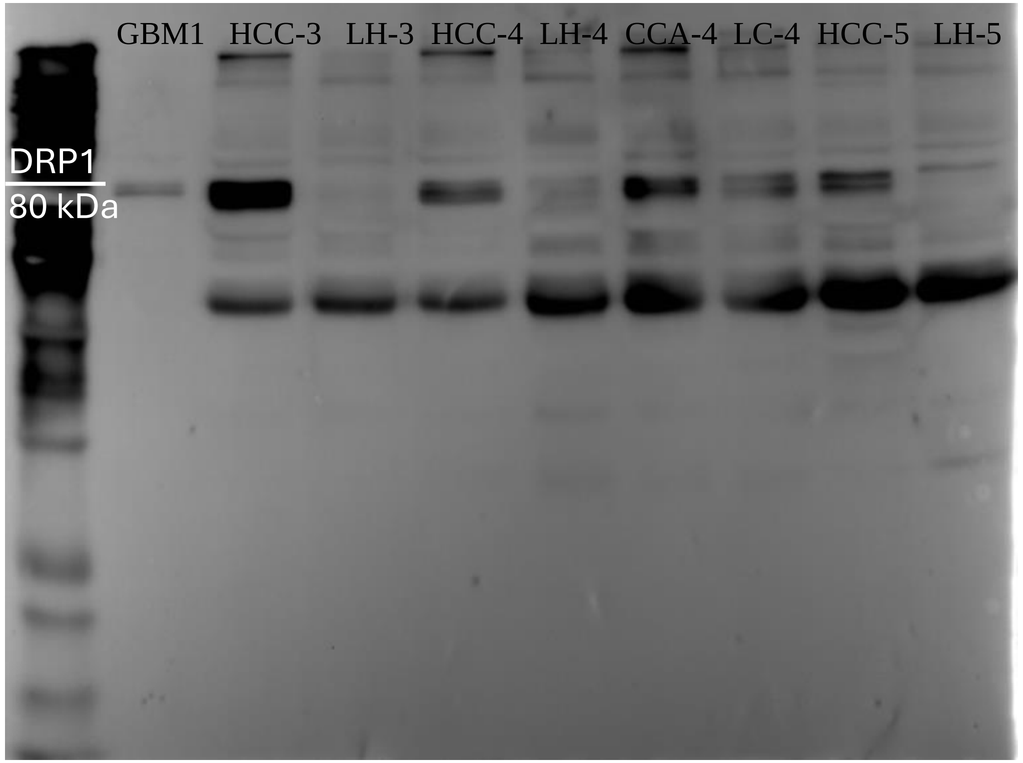

Figure 2C: actin

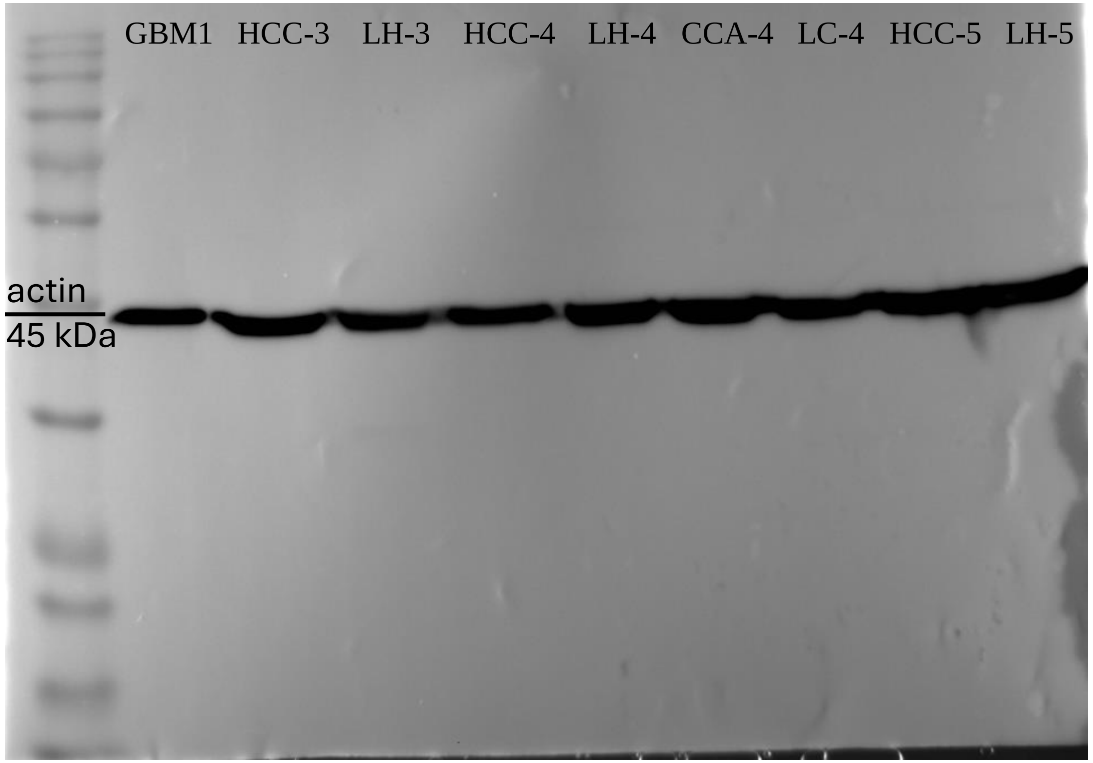

Supplement: Supplementary file 1 — Supplementary Material 1 [file 41598_2025_16379_MOESM1_ESM.pdf]
