# Supplementary material for: ZEB1 stratifies the response to Sorafenib and Mdivi-1 combination therapy in hepatocellular carcinoma
Source: Sci Rep. 2025 Aug 19;15:30451. doi: 10.1038/s41598-025-16379-6 (PMC12365315; doi:10.1038/s41598-025-16379-6)
Supplement: Supplementary file 2 — Supplementary Material 2 [file 41598_2025_16379_MOESM2_ESM.docx]

Supplementary Table 1:

Primers and antibodies used:

| **Target** | **Manufacturer / Order No.** | **Concentration** |
| --- | --- | --- |
| DRP1 | Proteintech; 12957-1-AP | 1:5.000 |
| ZEB1 | Sigma-Aldrich HPA027524 | 1:5.000 |
| ß-Actin | Santa-Cruz; sc-47778 | 1:1.000 |
| mouse-anti-rabbit HP conjugated sec. antibody | Santa-Cruz; sc-2357 | 1:120.000 |
| Goat-anti-mouse DyLight 649 | Rockland; 610-143-007 | 1:50.000 |

Supplementary table 2: Primers ordered from MicroSynth

| **Target** | **Sequence** |
| --- | --- |
| ZEB1_F | 5'-CGA ACC CGC GGC GCA ATA-3' |
| ZEB1_R | 5'-CCA GCA GTT CTT AGC ATT CC-3' |
| DRP1_F | 5'-CTG CCT CAA ATC GTC GTA GTG-3' |
| DRP1_R | 5'-GAG GTC TCC GGG TGA CAA TTC-3 |
| ß-Actin_F | 5’-CGA CAG GAT GCA GAA GGA G- 3’ |
| ß-Actin_R | 5’- ACA TCT GCT GGA AGG TGG A- 3’ |
| E-Cadherin_ F | 5’-CCCGGGACAACGTTTATTAC-3’ |
| E-Cadherin_R | 5’-GCTGGCTCAAGTCAAAGTCC-3’ |
| N-Cadherin_F | 5’-ACAGTGGCCACCTACAAAGG-3’ |
| Fibronectin_F | 5’-TCCCTCGGAACATCAGAAAC-3’ |
| Fibronectin_R | 5’-CAGTGGGAGACCTCGAGAAG-3’ |
| Vimentin_F | 5’-TACAGGAAGCTGCTGGAAGG-3’ |
| Vimentin_R | 5’-ACCAGAGGGAGTGAATCCAG-3’ |
| LGR5_F | 5’-TCA GTC AGC TGC TCC CGA AT-3’ |
| LGR5_R | 5’-CGT TTC CCG CAA GAC GTA AC-3’ |
| CK19_F | 5’-GCA CTA CAG CCA CTA CTA CAC GA-3’ |
| CK19_R | 5’-CTC ATG CGC AGA GCC TGT T-3’ |
| EPCAM_F | 5’-GAC TTT TGC CGC AGC TCA GGA-3’ |
| EPCAM_R | 5’-AGC AGT TTA CGG CCA GCT TGT-3’ |

Supplementary Table 3:

Cancer cell lines used in this study and a basic outline of their characteristics.

| **Cell line** | **Tumor entity** | **Patient data** | **Mutations** | **Doubling time** | **Other** |
| --- | --- | --- | --- | --- | --- |
| Huh7 | HCC | 57Y, male, japanese | TERT, TP53 | ~ 22hrs |  |
| HepG2 | HCC | 15Y, male, argentinian | TERT | ~ 48hrs |  |
| snu475 | HCC | 43Y, male, korean | TP53, TERT | ~ 66hrs | Hepatitis B (+) |
| snu387 | HCC | 41Y, female, korean | NRAS, TP53 | ~ 61hrs | Hepatitis B (+) |
| snu423 | HCC | 40Y, male, korean | TERT, TP53 | ~ 72hrs | Hepatitis B (+) |
| HuCC-T1 | CCA | 56Y, male, japanese | KRAS, MSH6, TP53 | ~ 74hrs |  |
| HuCC-A1 | CCA | 54Y, male, thai |  | ~ 55hrs |  |
| TFK-1 | CCA | 63Y, male, japanese | TP53 | ~ 37hrs |  |
